# In-Depth Profiling of T-Cell Responsiveness to Commonly Recognized CMV Antigens in Older People Reveals Important Sex Differences

**DOI:** 10.3389/fimmu.2021.707830

**Published:** 2021-08-13

**Authors:** Bernhard Reus, Stefano Caserta, Martin Larsen, George Morrow, Aalia Bano, Michael Hallensleben, Chakravarthi Rajkumar, Alejandra Pera, Florian Kern

**Affiliations:** ^1^Department of Informatics, School of Engineering and Informatics, University of Sussex, Brighton, United Kingdom; ^2^Department of Biomedical Sciences, The University of Hull, Hull, United Kingdom; ^3^Sorbonne Université, Inserm U1135, Centre d’Immunologie et des Maladies Infectieuses (CIMI-Paris), Paris, France; ^4^Department of Clinical and Experimental Medicine, Brighton and Sussex Medical School, Brighton, United Kingdom; ^5^Institute for Transfusion Medicine and Transplant Engineering, Hannover Medical School, Hannover, Germany; ^6^Immunology and Allergy Group, Maimonides Institute for Biomedical Research of Cordoba (IMIBIC), Córdoba, Spain; ^7^Department of Cell Biology, Physiology and Immunology, University of Córdoba, Córdoba, Spain

**Keywords:** aging, T cell, Cytomegalovirus (CMV), immunosenescence, biological sex

## Abstract

The impact of biological sex on T-cell immunity to Cytomegalovirus (CMV) has not been investigated in detail with only one published study comparing CMV-specific T-cell responses in men and women. Many studies, however, have shown an association between CMV infection and immunosenescence, with broad effects on peripheral blood lymphocyte subsets as well as the T and B-cell repertoires. Here, we provide a detailed analysis of CMV-specific T-cell responses in (n=94) CMV+ older people, including 47 women and 47 men aged between 60 and 93 years. We explore sex differences with respect to 16 different CMV proteins arranged in 14 peptide pools (overlapping peptides). Following ex vivo stimulation, CD4 and CD8 T-cells producing IFN-γ, TNF, and IL-2 were enumerated by flow-cytometry (intracellular cytokine staining). T-cell responses were evaluated in terms of each cytokine separately or in terms of cytokines produced simultaneously (polyfunctionality). Surface memory phenotype and CD3 downmodulation were assessed in parallel. The polyfunctionality index and a memory subset differentiation score were used to identify associations between response size, cytokine production, polyfunctionality, and memory subset distribution. While no significant sex differences were found with respect to overall CMV target protein selection, the T-cell response in men appeared more focused and accompanied by a more prominent accumulation of CMV-specific memory CD4 and CD8 T-cells. T-cell polyfunctionality and differentiation were similar in the sexes, however, CMV-specific T-cells in men produced more pro-inflammatory cytokines. Particularly, TNF production by CD4 T-cells was stronger in men than in women. Also, compared with women, men had larger responses to CMV proteins with immediate-early/early kinetics than women, which might have been driven by CMV reactivation. In conclusion, the CMV-specific T-cell response in men was larger and more pro-inflammatory than in women. Our findings may help explain sex differences in CMV-associated pathologies.

## Introduction

Cytomegalovirus (CMV) infection has a major impact on the immune system, in particular on the distribution of lymphocyte subsets (1, 2). CMV prevalence increases with age, which also has a profound effect on the immune system by reducing its ability to respond to infection and cancer (immunosenescence). This CMV prevalence results in increased morbidity and mortality in older people. Biological sex appears to modify both the effect of CMV infection and aging (3–6). It is important to note that immunosenescence also occurs in the absence of CMV infection, however, changes resulting from CMV infection may accelerate the effects of immunosenescence.

A role of CMV infection in immunosenescence has been supported by numerous publications since the early 2000s. Loss of the costimulatory molecule CD28 on CD4 T-cells (generating ‘CD28^null^ CD4 T-cells’) has been recognized as a hallmark of immune ageing since 1999 (7) and for just as long, it has been clear that CMV is associated with its occurrence (8). This culminated in the hypothesis that immunosenescence may be ‘infectious’ (9). Our own recent work (10) has clarified that CD28^null^ CD4 T-cells do not increase with age among CMV-seronegative (CMV-) people showing that accumulation of these cells is not related to aging *per se*. So, aging and immune aging are not the same thing, they just coincide in older people. CD28^null^ CD4 T-cells clearly have features of ‘aged’ immune cells (e.g., replicative senescence) and the immune system of people with expanded CD28^null^ CD4 T-cells would, therefore, appear more aged. Since older people tend to be more frequently CMV-seropositive (CMV+) than younger individuals, the effects of aging and CMV cannot be easily differentiated in the elderly. CMV prevalence can be up to 90% or more in many parts of the world (11).

CMV has been implicated in memory T-cell inflation, in particular in mouse models (12), and in a number of conditions associated with changes to the peripheral blood T-cell compartment, including autoimmune disease, atherosclerosis, and cardiovascular disease (CVD). CVD is not only more common in older ages, but also shows considerable sex bias (13, 14). Our own recent work demonstrated an association of CMV infection with increased aortic stiffness in healthy, White British older males (15). Although confirmation of these findings in populations of different genetic backgrounds and social circumstances is pending, our findings suggest a potentially very important effect of biological sex on CMV-induced immunopathology. However, despite some inroads, detailed knowledge of sex-related differences in CMV-associated immunity and immunopathology is lacking. A number of studies have established that CMV infection biases the differentiation of the overall T-cell repertoire (2, 3, 6), however, it is still unclear to what extent CMV-specific T-cells may account for this effect. The Berlin Aging Study II (BASE-II) showed that both age and CMV infection drive T-cell differentiation, in both the CD4 and CD8 compartments, with men accumulating higher frequencies of terminally differentiated CD57+ CD8 T-cells. These cells are strongly associated with CMV infection and have high cytotoxic potential (3). Overall, older people had significantly higher proportions of late-differentiated TEMRA cells (T effector memory cells re-expressing CD45RA) among both men and women, but, interestingly, these were exclusively accounted for by CMV+ subjects. Our own recent study on vascular stiffness also suggested a stronger effect of CMV on memory T-cell differentiation in men than in women, which could be one possible explanation for increased vascular stiffness in CMV+ older men (15).

To our knowledge, the only published study on sex differences with respect to CMV-antigen specific T-cell responses dates back to 2004 (16). It showed that the *in vitro* CD4 T-cell response to a CMV virus lysate was dominated by IFN-γ and that women exhibited higher levels of IL-2, IL-2-secreting cells, and proliferation than men, suggesting that women react more strongly to CMV than men. Since T-cells are the mainstay of CMV-specific immunity, it is surprising that such differences have not been explored in more detail since. In this study, we use overlapping peptide pools for T-cell stimulation rather than a virus lysate. This has a number of advantages, not least the fact that both CD4 and CD8 T-cells can be stimulated efficiently (17, 18). A landmark study on CMV-specific T-cell immunity published in 2005 showed that this approach is very powerful (19). In that study CD4 and CD8 T-cell responses to 213 different CMV proteins (ORFs) were tested in 33 adults of different ages and biogeographical ancestries. This historic dataset was later reanalyzed to gain insight into the variability of CMV-specific T-cell immunity in different individuals (20), but the cohort was too small to robustly address sex differences. Our current dataset, however, is ideally suited for this purpose; the population of n=94 individuals of at least 60 years of age is evenly split into women and men, which ensures not only that the effects of CMV on the immune system are visible in most participants, but also that comparisons between the sexes are statistically robust. Our work focuses on the most important CMV target proteins in order to highlight patterns of responsiveness, including response size, differentiation, (poly-)functionality, and efficiency of activation (CD3 downmodulation). This study is the first ever to show a range of significant sex differences in CMV-specific T-cell immunity that will help explain different immunopathologies in women and men following CMV infection.

## Materials and Methods 

### Ethics Statement

Study approved by the UK National Research Ethics Service (NRES) London Centre (Reference 13/LO/1270). Written informed consent was obtained from all participants. The study was conducted in accordance with the Declaration of Helsinki.

### Participants and Samples

Generally healthy (n=94), CMV+ older volunteers (60-94 years) were recruited through general practices (GP) in Southern England with help of the primary care research network (PCRN). Individuals with previous vascular events, such as TIA, stroke or CV complications were included if generally well with normal physical activity. However, individuals with advanced CV morbidity were excluded.

Inclusion criteria were: White British ethnicity and age 60 years or older; exclusion criteria were: known immunodeficiency (including HIV infection), organ transplantation, use of immunosuppressive or immune-modulating drugs within the last year (excluding acetylsalicylic acid ≤ 100mg/day), cancer or treatment for cancer within the previous 5 years, insulin dependent diabetes, moderate or advanced renal failure, liver disease, endocrine disorders (except corrected thyroid dysfunction), manifest autoimmune disease, dementia/mental incompetence, known alcoholism or other drug abuse, acute infection or illness in the last 4 weeks, raised body temperature (>37.5°C), moderate or severe heart failure (NYHA III or IV), inability to lie flat. Individuals with typical, age-related cardiovascular morbidity were not excluded.

### Participant Data and Sample Collection

Peripheral blood from each subject was collected by venipuncture in sodium Heparin-containing tubes. PBMCs were isolated after collection using Ficoll-Hypaque density gradient centrifugation (Sigma Aldrich, Steinheim, Germany).

### CMV Serology

CMV immunoglobulin G (IgG) serology (Architect CMV IgG, Abbot, Maidenhead, UK) was performed at the Brighton and Sussex University Hospital Trust (BSUHT) virology laboratory.

### CMV Peptides

Overlapping peptide pools representing 16 CMV proteins arranged in 14 pools (**Table 1**) were used in T-cell stimulation assays [for details see **Supplementary Material** and (15)].

**Table 1:** CMV protein-covering peptide-pools used for T-cell stimulation^a^.

Pool	CMV Protein(s)	Kinetic class^c^ (21)	No. of Peptides
**1**	none	n.a.	n.a.
**2**	UL55	L	224
**3**	UL83 (‘pp65’)	L	138
**4**	UL86	E-L	340
**5**	UL122 (‘IE2’)	IE (L)	120
**6**	UL123 (‘IE1’)	IE	143
**7**	UL153	L	67
**8**	UL32	L	260
**9**	UL28	L	92
**10**	UL48A^b^	L	281
**11**	UL48B^b^	L	281
**12**	US3	IE	44
**13**	UL151& UL82	unclassified & L	219 (82 &137)
**14**	UL94 & US29	L&E-L	197 (84 &113)
**15**	US24 & UL36	E&E(IE)	240 (123 &117)
**16**	SEB (positive control)		

^a^A panel of 19 CMV protein-spanning peptide pools was previously shown to correlate highly with the CD4 and CD8 T-cell response against 203 tested CMV proteins (19). The original panel contained UL99, UL103, and US32 in addition, but were left out here since responses were absent in >100 White British people. ^b^UL48 was divided into two pools (UL48A and UL48B), however, results were combined with respect to determining T-cell reactivity. ^c^IE, immediate early; E, early; L, late; n.a., not applicable.

### CMV Reactivity of T-Cells

PBMCs were stimulated overnight (16h) with CMV peptide pools. The following morning cells, were stained and acquired by flow-cytometry. Phenotype markers included CD3, CD4, CD8, CD45RA and CCR7 (surface staining). Activation markers included IL-2, TNF, and IFN-γ (ICS). The gating strategy is shown in **Figure S1**. Responses were considered positive when a clustered population of activated cells was identified (visual inspection) above 0.01% of CD4 or CD8 T-cells after background subtraction from the unstimulated sample. For more details, see **Supplementary Material** and (15).

### T-Cell Counts

T-cell counts per nanoliter (nL) of blood were determined using a dual platform approach. White blood counts were obtained from the routine clinical laboratory using a Sysmex counter (Sysmex, UK). Using fresh whole blood, CD45+ cells (white blood cells) were gated as well as CD4 and CD8 T-cells (using CD3, CD4, and CD8). CD4 and CD8 T-cells per nL of blood were determined in two steps. First, the proportion of CD4 and CD8 T-cells among WBC was determined by dividing the CD4 and CD8 T-cell counts in the CD45+ gate (absolute event count) by the CD45+ gate count (absolute event count). This proportion was then multiplied with the WBC. Absolute CD4 and CD8 T-cell counts were available in 47 men (100%) and 42 women (89.4%).

### Data Processing

The raw data obtained from Flow Cytometry (FlowJo v9.6) was processed by a php script running PHP Version 7.1.33. The data processing involves the computation of percentage counts for each patient and tube, background subtraction, sums of subset counts for each patient (and type of subset), and the production of FunkyCells compatible data files, containing percentages for each T-cell memory subset.

### HLA Typing

HLA-typing was performed at the Institute for Transfusion Medicine, Hannover Medical School, Hannover/Germany [see (10)].

### Polyfunctionality Index and Differentiation Score

Polyfunctionality analysis was performed using FunkyCells software (www.Funkycells.com). The polyfunctionality index was described previously (22). Of note, here we performed the analysis on antigen-specific T-cells by gating on cells secreting at least one cytokine.

$$\text{Polyfunctionality Index }=\Sigma_{i=0}^{k}\;F_{i}*\frac{i}{k},$$

where *k* is the total number of functions studied, *F_i_* is the frequency (%) of cells performing *i* functions.

The differentiation profiles of T-cells were based on CD45RA and CCR7 expression. The four differentiation states were identified as Naive (i=0, CD45RA+CCR7+), Central Memory (i=1, CD45RA-CCR7+), Effector Memory (i=2, CD45RA-CCR7+) and Revertant (i=3, CD45RA+CCR7-).

$$\text{Differentiation Score }=\Sigma_{i=0}^{3}\;\%Subset*\frac{i}{3}$$

### Statistical Analysis

Sidak’s (biological sex) and Tukey’s (CMV proteins) multiple comparison correction were applied for multiple end-point correction with respect to gender and protein-specific T-cell reactivity, and gender and protein-specific CD3 downregulation in 2-way ANOVA analyses.

### Charts

The 3D bar charts were created by a Python program running Python version 3.3.7. The following python libraries were used: numpy, pandas, matplotlib, math, and scipy.stats. Scatter pie charts were generated using R (version 3.6.2) (23) and the packages ggplot2 (24) and scatterpie (25).

## Results

### Global Distribution of CMV Protein Reactivity Does Not Differ Between Males and Females

We recently recruited a cohort of 108 CMV seropositive and 101 CMV seronegative individuals between 60 and 93 years of age (10, 15). The present analysis comprises 94 CMV+ participants, in whom T-cell responses to CMV were measured including 47 women and 47 men of very similar age distribution (70.7 ± 6.5 years and 71.1 ± 8.2 years, respectively, mean ± SD). For testing T-cell reactivity to CMV, we used overlapping peptide pools covering 16 CMV proteins arranged in 14 stimulation pools (**Table 1**). The 16 proteins were selected from a group of 19 proteins previously identified to correlate highly with the T-cell response to all CMV proteins (19), but three proteins previously not inducing a single response in >100 White British individuals were omitted (26). Note that the selected proteins cover different kinetic classes (**Table 1**), which are being considered as part of the analysis. The division of proteins by kinetic classes accounts for the fact that different CMV proteins occur at different times after CMV reactivation. More precisely, those detectable at the earliest times are referred to as ‘immediate early’ (IE) and those that appear last are part of the virion at late (L) times. Some proteins appear in between, i.e., at early (E) and early-late (E-L) times (21, 27).

**Figure 1:**
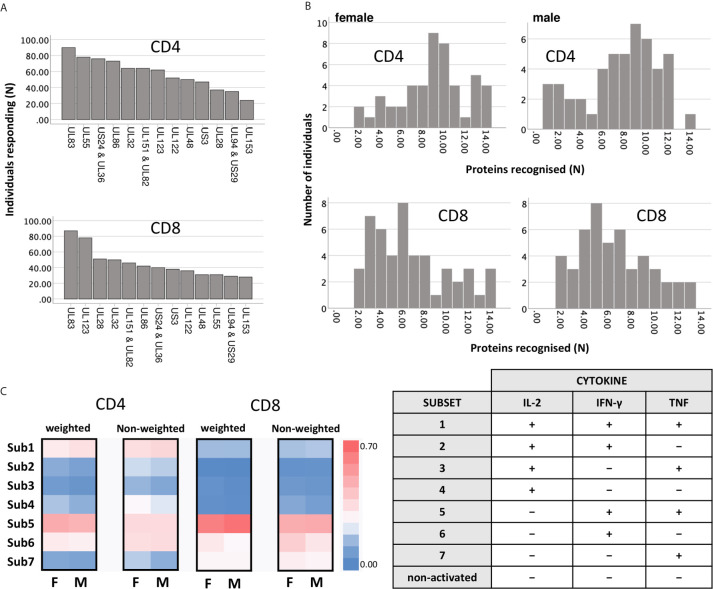
Reactivity of CD4 and CD8 T-cells against dominant CMV proteins. **(A)** The indicated proteins (overlapping peptide pools) were used for PBMC stimulation. Responses (IL-2, TNF, or IFN-γ) equal to or higher than 0.01% (1/10,000) of the reference population (CD4 or CD8) were included. The graph shows the number of individuals recognising the indicated CMV protein pools within the entire cohort (n=94). **(B)** The graph shows the number of recognised proteins per individual (x-axis) in females (right panels, n=47) and males (left panels, n=47), respectively. **(C)** Heatmaps show the frequency distribution of functional Subsets 1 to 7 (respectively: IL-2+/TNF+/IFN-γ+; IL-2+/TNF+/IFN-γ-; IL-2+/TNF-/IFN-γ+; IL-2+/TNF-/IFN-γ-; IL-2-/TNF+/IFN-γ+; IL-2-/TNF+/IFN-γ-; and IL-2-/TNF-/IFN-γ+, right table) within CD4 and CD8 T-cells, in females and in males. The heatmaps on the left show the contribution of each subset after weighing this relatively to the overall response size, while the heatmaps on the right show the unweighted contribution (not normalised for the response size).

For the representation of protein-specific reactivity, the responses to two pools including UL48 peptides (UL48A and UL48B) were combined (**Figure 1**). We first analyzed CD4 and CD8 T-cell responses to each pool with respect to the three cytokines measured (IL-2, TNF, IFN-γ). This was done cytokine by cytokine and in combination. (**Figure S1**, gating strategy). Logical combination of two possible states for each cytokine (i.e., IL-2+ or IL-2-, TNF+ or TNF-, IFN-γ+ or IFN-γ-) resulted in seven non-overlapping (Boolean) subsets of cells with one or more positive markers (**Figure 1C**, 1-7 hereafter), and one non-activated all-negative subset (IL-2-/TNF-/IFN-γ-). Individuals were considered ‘reactive’ to a pool if the percentage of activated events was equal to or larger than 0.01% (1/10,000) within CD4 and CD8 T-cells. After ranking the responses according to the number of individuals reacting to each CMV protein pool, the top five CD4 T-cell targets (UL83, UL55, US24 & UL36, UL86 and UL32) and top two CD8 T-cell targets (UL83 and UL123) were the same as in our previous study (28), but the order deviated slightly for the less dominant proteins (**Figure 1A**). The number and frequency distribution of protein target-specific T-cell responses were very similar in the two sexes (**Figure 1B**). For CD4 T-cells the median number of recognized proteins was 9 (IQR 7-11) in women and 8 (IQR 6-10) in men, the difference being borderline significant (*p*=0.082), for CD8 T-cells it was 6 (IQR 4-9) in both groups.

Theoretically, CMV protein recognition by T-cells should be affected by HLA-type. Peptides arising from a given CMV target protein will bind to some, but not all HLA-alleles. Individuals possessing the right alleles are, therefore, more likely to respond to these peptides than other individuals. All the participants of this study were White British and no significant difference between men and women in terms of target protein recognition was observed (**Figure 1B**). Regarding protein-specific response size, we observed a statistically significant difference between men and women in the size of the CD8 T-cell response to UL123 (IE-1) and UL153 proteins (*p ≤* 0.05/14, i.e., *p ≤* 0.0036, Bonferroni correction for 14 endpoints), which could have been caused by differences in the frequencies of one or more class-I HLA-alleles. HLA-typing (2-field-code) was available for only 28 women and 25 men. The difference with respect to these protein-specific responses, however, was not significant among the HLA-typed individuals, so that a possible effect of HLA-type on these responses could not be explored. As would be expected in a group of this size (given the vast polymorphism of the HLA-locus), HLA-alleles were not distributed completely evenly between women and men. Among the 53 HLA-typed participants, a total of 109 class-I and class-II HLA-alleles were present. Using the CHI^2^ test (Fisher’s exact test), we identified one single (class-II) HLA allele that was distributed significantly differently between the sexes at the *p ≤* 0.05 level (HLA-DQB1*03:01, *p*=0.02, CHI^2^ test). Multiple end-point correction, however, would reduce the significance level to *p ≤* 0.05/109, i.e., *p*≤0.00046. No allele difference between the sexes was significant at that level. On the whole, the number of HLA-typed individuals was too small to allow a rigorous analysis of the effect of HLA-type on protein-specific T-cell response sizes in women and men.

Next, the distribution of functional subsets 1 to 7 across all CMV proteins by size (**Figure 1C**) was determined either as weighted (contribution is proportional to protein-specific response sizes) or unweighted (all responses make the same contribution). No significant sex differences in functional subset distribution were detected at this level.

### Among Men, Response Profiles Are More Focused on Fewer Dominant Responses Than in Women

To explore potential sex differences, we conducted an in-depth analysis of T-cell responsiveness to a range of known and dominant target proteins (**Table 1**) using *ex vivo* stimulation followed by intracellular cytokine detection. First, we investigated whether the overall CMV-specific T-cell response size differed between the sexes. This was achieved by summing up all CMV protein-specific responses (i) with respect to each cytokine separately (IL-2, TNF and IFN-γ) and (ii) all cytokines in combination. Although men generally showed higher (median) responses, no statistically significant sex differences were detected with respect to the overall CD4 T-cell response. However, CD8 T-cell responses measured in percent of CD8 T-cells were significantly bigger in men than in women with respect to TNF, IFN-γ, or ‘any’ activation marker. This was also true (and included IL-2) when considering T-cell counts per volume of blood (**Table 2**). Note that for the purpose of analyzing T-cell response size, we mostly rely on subset percentages in terms of CD4 and CD8 T-cells, which agrees with our own previous work and most reports in the literature. The percentage reflects the degree of commitment of the overall resource (CD4 and CD8 T-cells) to certain phenotypic/functional subsets, making them amenable to comparison. We have, however, also performed analyses in terms of cell counts per volume of blood. Overall, the two measures show a very high correlation (**Figure S2** and **Table S1**).

**Table 2:** Overall CMV-specific response size in men and women.

Parameter	All	Women	Men	Women *vs* Men
	Median (IQR)	Median (IQR)	Median (IQR)	*p* ^a^
**CD4 IL-2 (%)**	0.65 (1.34)	0.60 (0.97)	1.21 (1.73)	0.069
**CD4 TNF (%)**	2.42 (3.61)	2.28 (2.34)	3.08 (5.86)	0.192
**CD4 IFN-γ (%)**	2.17 (2.78)	2.06 (2.27)	2.23 (5.39)	0.268
**CD4 (any) (%)**	2.84 (3.81)	2.59 (2.53)	3.27 (6.43)	0.143
**CD8 IL-2 (%)**	0.29 (0.52)	0.20 (0.56)	0.35 (0.63)	0.128
**CD8 TNF (%)**	5.55 (6.64)	3.82 (5.90)	7.26 (7.91)	**0.008**
**CD8 IFN-γ (%)**	5.63 (7.26)	3.68 (6.56)	7.29 (7.22)	**0.004**
**CD8 (any) (%)**	6.53 (8.61)	4.10 (7.55)	8.61 (9.21)	**0.008**
**CD4 IL-2 (cells/nl)**	0.50 (0.98)	0.45 (0.59)	0.68 (1.11)	0.324
**CD4 TNF (cells/nL)**	1.74 (2.54)	1.83 (1.85)	1.70 (3.27)	0.593
**CD4 IFN-γ (cells/nL)**	1.29 (2.04)	1.53 (1.46)	1.18 (3.13)	0.721
**CD4 (any) (cells/nL)**	1.95 (2.69)	2.02 (1.75)	1.92 (3.59)	0.593
**CD8 IL-2 (cells/nL)**	0.09 (0.25)	0.08 (0.10)	0.14 (0.29)	**0.030**
**CD8 TNF (cells/nL)**	2.00 (3.25)	1.32 (3.00)	2.91 (3.26)	**0.006**
**CD8 IFN-γ (cells/nL)**	1.78 (3.43)	1.09 (2.88)	3.13 (3.40)	**0.005**
**CD8 (any) (cells/nL)**	2.39 (4.27)	1.56 (3.74)	3.61 (3.62)	**0.009**

a Mann-Whitney test, not corrected for multiple endpoints. Significant values (p≤0.05) are shown in bold.

We then investigated the question whether the size of the summated CMV-specific T-cell response correlated with the number of recognized CMV proteins in each individual. There was a weak, but statistically significant correlation among CD4 (R=0.258, *p*=0.012), but not CD8 T-cells (**Table S2**). The lack of a stronger correlation between these parameters reflected that the contribution of individual CMV protein-specific responses to overall response size was clearly highly variable (**Figures S3**, **S4**). The UL83 and UL55-specific responses among CD4 T-cells were significantly bigger than other responses. IL-2+ CD4 T-cell responses were dominated by responses to the late (L) structural target proteins, UL55, UL83, and UL86 (**Figure S3**). However, in regards to TNF and IFN-γ responses, the immediate early (IE) protein-specific responses also played a major role. US3 was slightly less frequently recognized than UL123 (IE-1) and UL122 (IE-2) (**Figure 1A**), yet clearly induced bigger CD4 T-cell responses (**Figure S3**). As previously reported (26, 28), the CD8 T-cell response was dominated by UL83 and UL123 (**Figure 1A** and **Figure S3**). Of note, we found that CD8 T-cell responses to UL28 were very large in some individuals, but more variable than responses to UL83 and UL123. Essentially, the same patterns were revealed when using cell count per volume of blood (cells/nL) instead of percentages of CD4 or CD8 T-cells (**Figure S4**).

**Figure 2:**
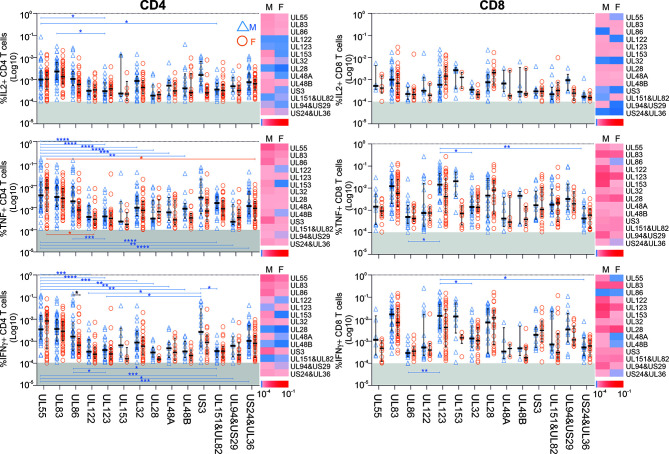
Impact of biological sex on the CD4 and CD8 T-cell reactivity against 14 different CMV peptide pools. PBMCs from males (n=47) and females (n=47) were stimulated with CMV peptide pools; responses below the 1/10,000 threshold were excluded (grey shaded area of each plot). Heatmaps represent response size in males (M) and females (F). As indicated in the scale, darker shades of red represent large responses, while darker shades of blue correspond to low response frequencies. Scatter plots show the frequencies of CD4 and CD8 T-cells responding to the indicated CMV proteins in males (blue empty triangles, M) and females (orange empty circles, F). Error bars show median and interquartile range. Statistical differences in response sizes with respect to different CMV protein were assessed in 2-way ANOVA (addressing both the effect of ‘sex’ and ‘CMV protein’ on the size of the cytokine response, **Table S3**) followed by multiple end-point correction. Differences between proteins within males and females are shown by blue and orange lines, respectively, and differences between males and females by black lines. Significance levels are: *p ≤ 0.05; **p ≤ 0.005; ***p ≤ 0.0005; ****p ≤ 0.0001.

We subsequently explored which proteins showed the biggest sex differences in response magnitude and if there were sex differences in regards to protein immunodominance (**Figure 2**). Overall, there was a clearly visible trend towards higher TNF and IFN-γ responses in men (**Figure 2**, heatmaps) both among CD4 and CD8 T-cells. However, using stringent multiple endpoint correction, only the difference in the size of the IFN-γ CD4 T-cell response to the UL86 protein was statistically significant. US3-specific CD4 T-cell responses were four to five times bigger in men than in women, but the difference did not reach statistical significance (**Figure 2**).

Interestingly, the CD4 T-cell response to UL55 differed significantly in size from responses to multiple other CMV proteins in men (blue) but not women (orange), underscoring the dominance of UL55 as CD4 T-cell target, particularly in men (**Figure 2**). In particular, with respect to TNF+ and IFN-γ+ CD4 T-cells, UL55-specific responses were significantly higher than responses to nine other proteins, but only in men. Also, UL123 CD8 T-cell responses were significantly higher than three other responses in men, but not in women. Together, these data suggest that protein recognition is more focused on dominant proteins in men than in women, with UL55, UL86 and US3-specific CD4 T-cell responses and UL123-specific CD8 T-cell responses contributing most to the overall larger summated response sizes.

We subsequently sought to investigate cytokine dominance in CMV-specific T-cell responses in the two sexes. In the entire cohort and with respect to each peptide pool, TNF and IFN-γ CD4 T-cell responses were on average higher than IL-2 responses (*p*<0.0001 for each comparison), and TNF responses were bigger than IFN-γ responses (Wilcoxon test, related samples, *p*<0.001). For CD8 T-cells, both TNF and IFN-γ responses were bigger than IL-2 responses (*p*<0.0001 for each comparison), however, the differences between TNF and IFN-γ responses were not statistically significant. All of the above differences persisted when men and women were analyzed separately.

### Sex Differences Are Reflected by CMV Target Protein-Associated CD3 Downregulation Patterns

With respect to most tested CMV-proteins, T-cell responses tended to be larger in men than in women (**Figure 2**). Consequently, we investigated if such differences were reflected by different degrees of activation-induced CD3 downmodulation, which is often used as a measure of stimulation efficiency. In each individual, CD3 downmodulation was assessed relative to the non-activated (i.e., cytokine-negative) portion of CD4 and CD8 T-cells. Because differences in cytokine production may be associated with different propensities to respond to antigenic challenge, we analyzed relative CD3 downmodulation in each of the seven functional subsets defined by IL-2, TNF, and IFN-γ production (**Figure 1C**). CD3 downmodulation was calculated as:

(MFI of the non‐responsive T‐cell population−MFI of the activated T‐cell population)/MFI of the non‐responsive T‐cell population

This typically resulted in values between 0 and 1 (‘1’ indicating 100% downmodulation). However, in a few cases, this ratio was negative (if the activated events had a higher MFI than the non-responsive population) or >1 (if the MFI of the activated T-cell population was below baseline, i.e., recorded with a negative value).

**Figure 3:**
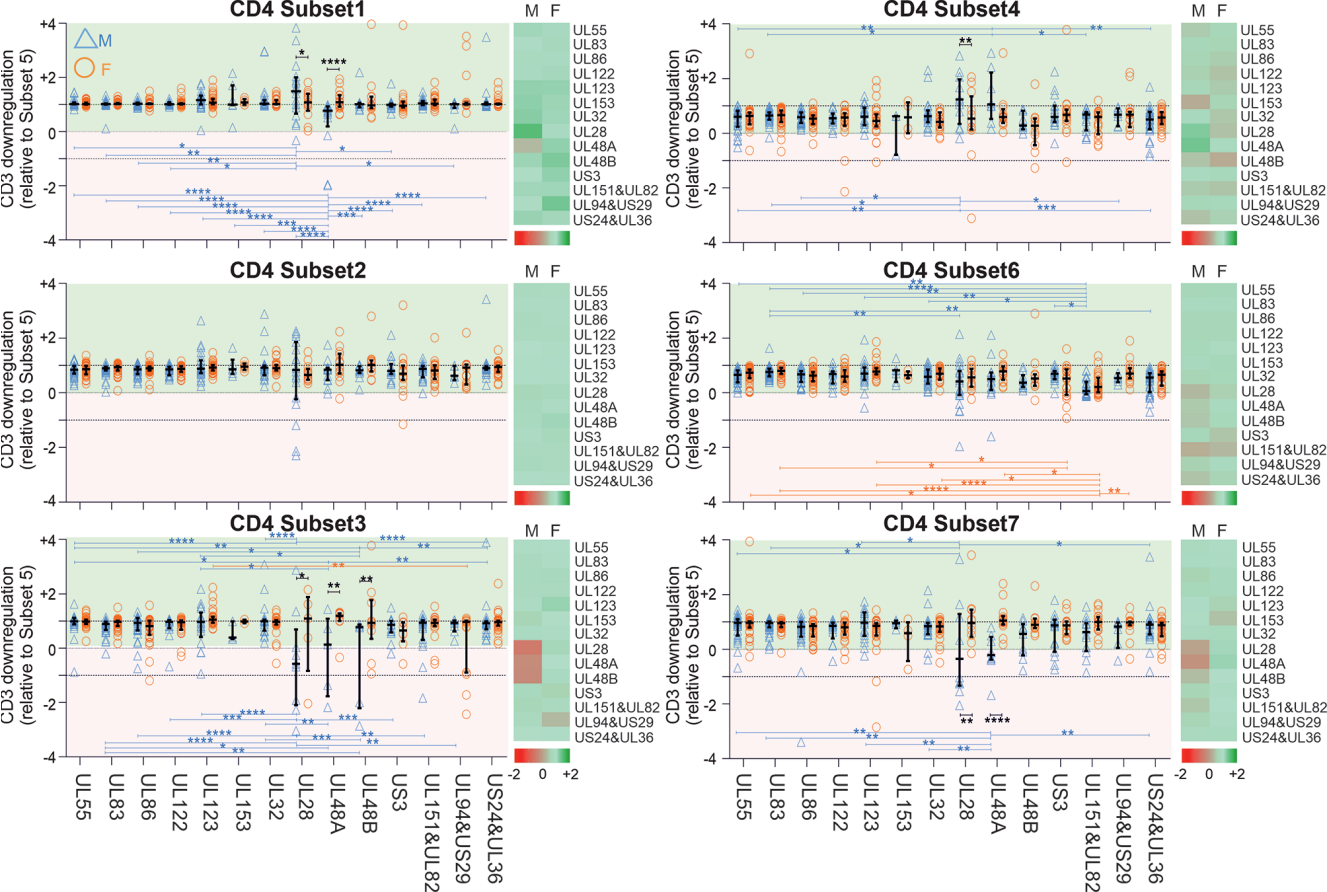
CD3 downregulation takes place to different degrees in different functional CD4 T-cell subsets and reveals sex differences in regards to select proteins. PBMCs from men (n=47) and females (n=47) were stimulated with specific CMV-protein peptide mixes and processed for intracellular cytokine staining, excluding responses below the 1/10,000 threshold. Levels of CD3 downregulation in functional subsets (Sub1 through Sub7) were standardized relative to the downregulation in Sub5. Heatmaps show CD3 downmodulation in males (M) compared with females (F). Darker shades of red represent low and darker shades of green high CD3 downmodulation. Scatter plots show CD3 downregulation in males (blue triangles) and females (orange circles). Error bars show median and interquartile range. Statistically significant differences (i) between sexes are indicated by black lines and (ii) within sexes between proteins by blue and orange lines for men and women, respectively. Statistical significance was determined by 2-way ANOVA followed by multiple end-point correction. Significance levels are *P ≤ 0.05; **P ≤ 0.005; ***P ≤ 0.001; ****P ≤ 0.0001.

Interestingly, CD3 downregulation varied significantly between the seven functional subsets in regards to each protein, but also the responses to different proteins. It also varied between women and men (**Figures 3** and **S5, S6**). Functional subset 5 (IL-2-/TNF+/IFN-γ+, ‘Sub5’) showed the biggest CD3 downmodulation of all subsets both in CD4 and CD8 T-cells (**Figure S5**). CD3 downmodulation in Sub5 was frequently close to 100% in CD4 T-cells but somewhat less often in CD8 T-cells. The heatmaps in **Figure S5** show a tendency towards lesser CD3 downmodulation in Sub5 in men than in women (dominance of red over green, **Figure S5**). CD3 downmodulation of Sub5 among UL28 and UL48A-specific CD4 T-cells was weaker than among CD4 T-cells specific to other proteins. With respect to CD8 T-cells, UL83 showing the highest levels of downregulation in both sexes, followed by UL55 and US3. No significant differences were elicited but, surprisingly, CD3 downmodulation overall was weaker in the CD8 T-cells (**Figure S5**).

Next, in order to establish if there was a hierarchy of CD3 downmodulation across the functional subsets, we standardized CD3 downmodulation across all subsets to the degree of CD3 downmodulation found in Sub5, which was the most downmodulated subset (i.e., the level of CD3 downmodulation of each subset was divided by that of Sub5) (**Figures 3** and **S6**). Relative CD3 downmodulation for each subset was hence between 0 and 1 with a very small number of exceptions in individuals where a subset other than Sub5 showed the strongest CD3 downmodulation (**Figures 3** and **S6**).

With respect to CD4 T-cells (**Figure 3**), Sub1 showed a degree of CD3 downmodulation similar to Sub5 (values close to 1, in general). In regards to the most immunodominant proteins, UL55, UL83 and UL86 (**Figure 2**), CD3 downmodulation was high in virtually all functional CD4 subsets in both men and women (**Figure 3** and **S6**). In CD8 T-cells, interestingly, CD3 downregulation across the functional subsets was less consistent and showed more heterogeneity across different target proteins (**Figures S5** and **S6**). **Table S4** shows *p*-values for differences in CD3 downmodulation between the functional subsets as well as between the sexes. These results agree with the sex differences reported in the previous sections. Generally, sex differences were more visible in CD4 than in CD8 T-cells. In men the CMV-specific immune response appeared more polarized towards dominant targets than in women.

### Men Accumulate More CMV-Specific CD4 and CD8 Memory T-Cells Than Women

**Figure 4:**
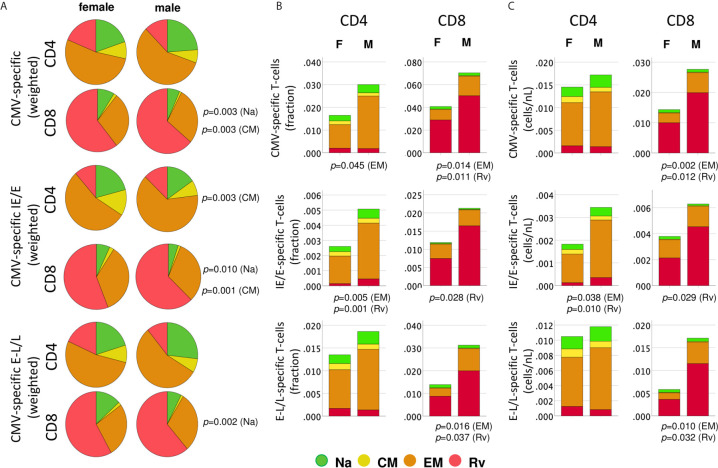
Distribution of protein-specific T-cells across canonical blood T-cell subsets by protein kinetic class. CMV-specific T-cells (any activation marker) were analyzed in regards to memory subset composition including naïve-like/stem-cell memory (Na, CCR7+CD45RA+, green), central memory, (CM, CCR7+CD45RA-, yellow), effector memory (EM, CCR7-CD45RA-, orange) and revertant memory (Rv, CCR7-CD45RA+, red). **(A)** Pie charts show weighted average percentages of memory T-cell subsets (composition). Top, middle, and bottom panels show all CMV-protein reactive T-cells, IE/E-protein-and E-L/L-protein-specific T-cells, respectively. Weighted percentages were obtained by adding up responder frequency for all selected proteins, and then the sums for each subset were normalized to 100%. **(B)** Stacked bar charts show non-normalized CD4 or CD8 T-cell subset percentages added up across all CMV proteins, IE/E and E-L/L proteins, visualizing overall subset size differences between men and women. **(C)** Stacked bar charts show non-normalized CD4 or CD8 T-cell counts/nL of blood added up across all CMV proteins, IE/E and E-L/L proteins, providing an alternative visualization of subset size differences between men and women. *p-*values indicate significant (*p ≤* 0.05) differences between females and males.

We wondered whether the biological sex differences detected above in cytokine responses were mirrored in the distribution of canonical peripheral blood T-cell subsets. The surface markers C-C chemokine receptor 7 (CCR7) and CD45RA were used to identify canonical T-cell ‘memory subsets’ including naïve/naïve-like T-cells (CCR7+CD45RA+, Na), central memory (CCR7+CD45RA-, CM), effector memory (CCR7-CD45RA-, EM), and revertant memory T-cell subsets (CCR7-CD45RA+, Rv) (**Figure S1**) (15). Of note, the ‘naïve’ phenotype will include a variable and generally small number of T-cells that are antigen experienced, but exhibit a phenotype similar to that of naïve cells, referred to as naïve-like cells (Na). The distribution of these four subsets among CMV-reactive T-cells was analyzed in women and men with respect to each protein/protein-combination pair (**Figure S7**) and the overall distribution (**Figure 4**).

We initially examined differences in the proportions of all memory subsets among CMV-specific T-cells, which may identify changes in response composition between the sexes (**Figure 4A**).

Importantly, to generate these diagrams, the percentages of each respective subset in each individual were first added up across all CMV proteins (top), immediate-early/early proteins (IE/E, middle), and early-late/late proteins (E-L/L, bottom) and then the sums were normalized to 100% for representation in the pie charts. Larger responses to some proteins, therefore, have a stronger effect on these pie charts than smaller responses and hence have more weight; we refer to these charts as ‘weighted’ (averaging the proportions across the different proteins in the first step, by contrast, would give each protein the same weight). Regarding all CMV-proteins together, the memory subset composition of CD4 T-cell responses did not significantly differ between women and men. With respect to CD8 T-cells, however, the Na and CM compartments were significantly larger in women than in men (**Figure 4A**, top). Significant sex differences were also found in regards to IE/E-specific CM CD4 T-cells, Na and CM CD8 T-cells, and E-L/L-specific Na CD8 T-cells (**Figure 4A**, middle and bottom). These results suggest that the proportions of the Na and/or CM CD8 T-cell (less differentiated) compartments in men were generally smaller. **Figure 4B** shows memory subset frequencies (summed percentages) in terms of CD4 and CD8 T-cells rather than response composition. With respect to the overall frequencies of CMV-specific peripheral T-cells (top), EM CD4 T-cells (*p*=0.045) as well as EM (*p*=0.014) and Rv (*p*=0.011) CD8 T-cells were significantly larger in men than women. In regards to IE/E protein-specific T-cells (middle), men showed significantly more EM (*p*=0.005) and Rv (*p*=0.001) CD4 T-cells and, in addition, Rv (*p*=0.028) CD8 T-cells. Regarding E-L/L proteins (bottom), men had higher EM (*p*=0.016) and Rv (*p*=0.037) CD8 T-cells. **Figure 4C** confirms the identified sex differences showing, however, median counts per volume of blood (cells/nL), instead of percentages. The Rv CD8 T-cell subset in particular was clearly bigger in men than in women, for example, with respect to E-L/L proteins it was 3.7 times bigger in men (**Table 3**). On the whole, when considering response size both in terms of percentages and counts per volume of blood, significant sex differences became apparent, particularly regarding the sizes of the EM and/or Rv compartments among CD4 and CD8 T-cells. EM and Rv CD4 T-cell responses to IE/E as well as EM and Rv CD8 T-cell responses to E-L/L proteins were between two- and threefold higher in men than in women (**Table 3**).

**Table 3:** Biological sex differences in memory subset size and distribution.

	Women	Men	Women/Men	
	Median (IQR)	Median (IQR)	Ratio	*P*
Na CD4 IE/E (%)	0.03 (0.06)	0.06 (0.09)	0.57	n.s.
CM CD4 IE/E (%)	0.03 (0.05)	0.03 (0.04)	0.92	n.s.
**EM CD4 IE/E** (%)	0.18 (0.28)	0.37 (1.16)	**0.49**	**0.005**
**Rv CD4 IE/E** (%)	0.01 (0.03)	0.05 (0.15)	**0.32**	**0.001**
Na CD8 IE/E (%)	0.03 (0.07)	0.03 (0.05)	0.90	n.s.
CM CD8 IE/E (%)	0.01 (0.04)	0.01 (0.03)	0.99	n.s.
EM CD8 IE/E (%)	0.32 (0.62)	0.37 (0.9)	0.88	n.s.
**Rv CD8 IE/E** (%)	0.68 (1.09)	1.24 (2.48)	**0.54**	**0.028**
Na CD4 E-L/L (%)	0.19 (0.35)	0.28 (0.61)	0.70	n.s.
CM CD4 E-L/L (%)	0.14 (0.16)	0.11 (0.22)	1.19	n.s.
EM CD4 E-L/L (%)	0.85 (1.73)	1.34 (2.67)	0.64	n.s.
Rv CD4 E-L/L (%)	0.17 (0.34)	0.14 (0.65)	1.25	n.s.
Na CD8 E-L/L (%)	0.13 (0.39)	0.13 (0.3)	1.01	n.s.
CM CD8 E-L/L (%)	0.02 (0.03)	0.03 (0.04)	0.75	n.s.
**EM CD8 E-L/L** (%)	0.34 (1.01)	0.89 (1.42)	**0.39**	**0.016**
**Rv CD8 E-L/L** (%)	0.79 (3.85)	1.88 (4.15)	**0.42**	**0.037**
Na CD4 IE/E (cells/nL)	0.02 (0.04)	0.04 (0.06)	0.61	n.s.
CM CD4 IE/E (cells/nL)	0.02 (0.04)	0.02 (0.03)	1.18	n.s.
**EM CD4 IE/E** (cells/nL)	0.13 (0.19)	0.25 (0.56)	**0.50**	**0.038**
**Rv CD4 IE/E** (cells/nL)	0.01 (0.02)	0.04 (0.08)	**0.36**	**0.010**
Na CD8 IE/E (cells/nL)	0.02 (0.02)	0.01 (0.03)	1.62	n.s.
CM CD8 IE/E (cells/nL)	0 (0.02)	0.01 (0.01)	0.82	n.s.
EM CD8 IE/E (cells/nL)	0.13 (0.25)	0.15 (0.44)	0.87	n.s.
**Rv CD8 IE/E** (cells/nL)	0.21 (0.69)	0.4 (1.14)	**0.52**	**0.029**
Na CD4 E-L/L (cells/nL)	0.16 (0.26)	0.19 (0.28)	0.86	n.s.
CM CD4 E-L/L (cells/nL)	0.11 (0.14)	0.09 (0.13)	1.26	n.s.
EM CD4 E-L/L (cells/nL)	0.65 (1.32)	0.82 (1.97)	0.80	n.s.
Na CD4 E-L/L (cells/nL)	0.12 (0.28)	0.08 (0.26)	1.50	n.s.
Rv CD8 E-L/L (cells/nL)	0.06 (0.1)	0.07 (0.13)	0.88	n.s.
CM CD8 E-L/L (cells/nL)	0.01 (0.01)	0.01 (0.02)	0.75	n.s.
**EM CD8 E-L/L** (cells/nL)	0.14 (0.25)	0.45 (0.69)	**0.31**	**0.010**
**Rv CD8 E-L/L** (cells/nL)	0.28 (1.62)	1.02 (2.06)	**0.27**	**0.032**

Statistical significances (p ≤ 0.05) are shown in bold.

n.s., non-significant.

### The Amount of Cytokine Produced by CMV-Specific T-Cells Is Higher in Men Than in Women

In addition to response size, we also examined the overall IL-2, TNF, IFN-γ secretion ‘potential’ of CMV-specific CD4 and CD8 T-cells. This was done in order to explore if certain proteins had more intrinsic inflammatory potential than others and to elicit additional potential differences between men and women. Note that, at the single cell level, the mean fluorescence intensity (MFI) of the cytokine detection antibody directly correlates with the amount of intracellular cytokine. However, at the population level, the average MFI of CMV protein-specific responses does not provide a good measure of cytokine production unless the number of cells producing the cytokine is taken into account. By multiplying the cell number (percentage of reference population) and MFI, a surrogate for the amount of produced cytokine can be calculated that is sometimes referred to as ‘integrated MFI’ (iMFI) (29). We, therefore, used the iMFI to compare responses between women and men in addition to response size alone. Also note that with respect to cytokine production, the MFI of different protein-specific responses cannot reasonably be added up in order to summarize the amount of cytokine produced overall. Because the iMFI is a surrogate for cytokine amount, however, it is permissible (and makes sense, mathematically) to add iMFIs across responses. This allows for the computation of cytokine production in response to several proteins. **Table 4** shows that, with respect to CD4 and CD8 T-cells and responses to all CMV proteins, the iMFI for TNF and IFN-γ tends to be higher in men than in women. These differences were statistically significant for CD8 T-cells only.

**Table 4:** iMFI for TNF and IFN-γ between biological sex groups.

Parameter	Women		Men		*p*
	Median	(IQR)	Median	(IQR)	
CD4 TNF	468.37	(715.33)	644.04	(1941.48)	0.228
CD4 IFN-γ	70.42	(64.24)	86.26	(156.47)	0.189
**CD8 TNF**	546.55	(1055.63)	1299.14	(1405.64)	**0.006**
**CD8 IFN-γ**	171.42	(382.69)	364.62	(414.93)	**0.004**

Statistical significances (p ≤ 0.05) are shown in bold

### CMV Reactivation Responses Are Bigger in Men and Associate With Bigger E-L/L Responses, But Not Vice Versa

**Figure 5:**
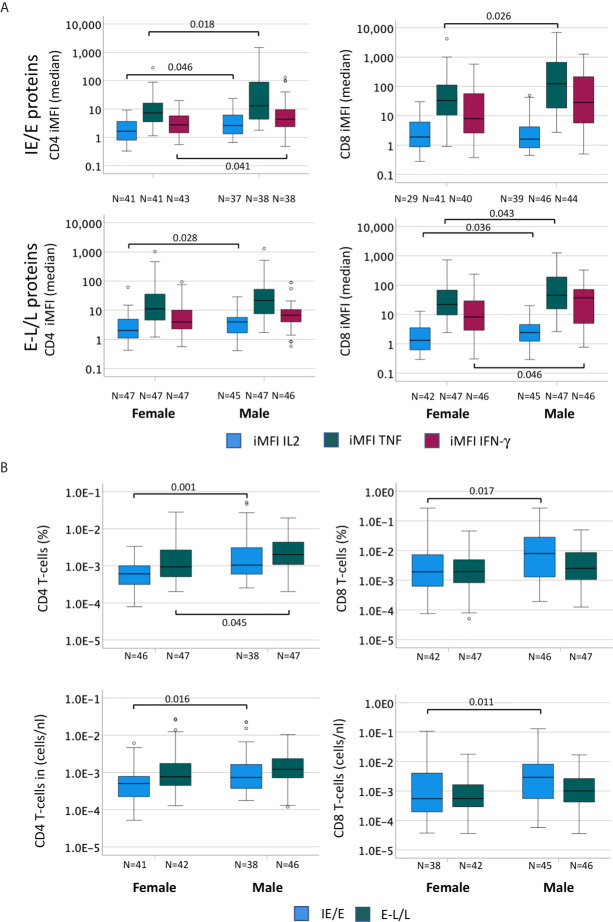
The cytokine content of CMV-specific T-cell responses is higher in men than in women. In order to compare the cytokine amounts produced by CMV-specific T-cells in men and women the percentage (in terms of CD4 or CD8 T-cells) of each cytokine response was multiplied by the MFI to generate the ‘integrated MFI’ (iMFI), for CD4 and CD8 T-cells. Responses were grouped by adding up the iMFI of responses to IE/E proteins (UL122, UL123, US3, US24&UL36), more likely to reflect CMV reactivation, and those to E-L/L proteins (UL55, UL83, UL32, UL28, UL48, UL151&UL83, UL94&US29). **(A)** Box plots show the median iMFIs for each cytokine response to IE/E (top) or E-L/L proteins (bottom) by CD4 (left) and CD8 (right) T-cells. **(B)** Box plots show the median of the summed frequencies or counts (cells/nL) of CD4 (left) and CD8 (right) T-cells responding to IE/E and E-L/L proteins. Box plots show median and interquartile range, outlier limits (whiskers, LQ-1.5*IQR, UQ+1.5*IQR), outliers (o). Within each graph, left and right boxes show responses in females and males, respectively.

CMV reactivation is a frequent event in CMV-infected people and thought to be probably the main driver of CMV-specific T-cell expansions (30). As a result, CMV reactivation is also likely to drive differences in CMV-specific T-cell responses between women and men. Accordingly, we examined differences between men and women regarding proteins whose expression is related to different times after activation. We hypothesized that proteins produced at immediate early and early times may have a bigger impact of shaping T-cell immunity than those produced later and that they may have different impact in women and men. We, therefore, calculated the median response iMFI (IL-2, TNF and IFN-γ) for all pools containing IE/E proteins (UL122, UL123, US3, US24&UL36) as well as E-L/L proteins (UL55, UL83, UL32, UL28, UL48, UL151&UL83, UL94&US29) and compared these between men and women (**Figure 5A**). Additionally, summated response sizes with respect to the same proteins (median) were compared between men and women (**Figure 5B**). The lower panel of **Figure 5B** shows counts per volume of blood, essentially confirming the results obtained with percentages. With respect to CD4 T-cells (**Figure 5A**, left), the median iMFI of the IL-2, TNF, and IFN-γ responses to IE/E proteins (top) was significantly higher in men than in women, as well as the iMFI of the E-L/L protein-specific IL-2 response (bottom). Regarding CD8 T-cells (**Figure 5A**, right), the median iMFI of the TNF response to IE/E proteins (top) as well as the median iMFI of IL-2, TNF, and IFN-γ responses to E-L/L proteins (bottom) were all significantly higher in men than in women. At the level of response sizes, CD4 T-cells response to IE/E and E-L/L proteins and CD8 T-cells response to E-L/L proteins were higher in men compared with women (**Figure 5B**). Interestingly, there was a moderate association between the size of the CD4 T-cell response to IE/E proteins and that to E-L/L proteins (any cytokine, R=0.325, *p*=0.003).

**Figure 6:**
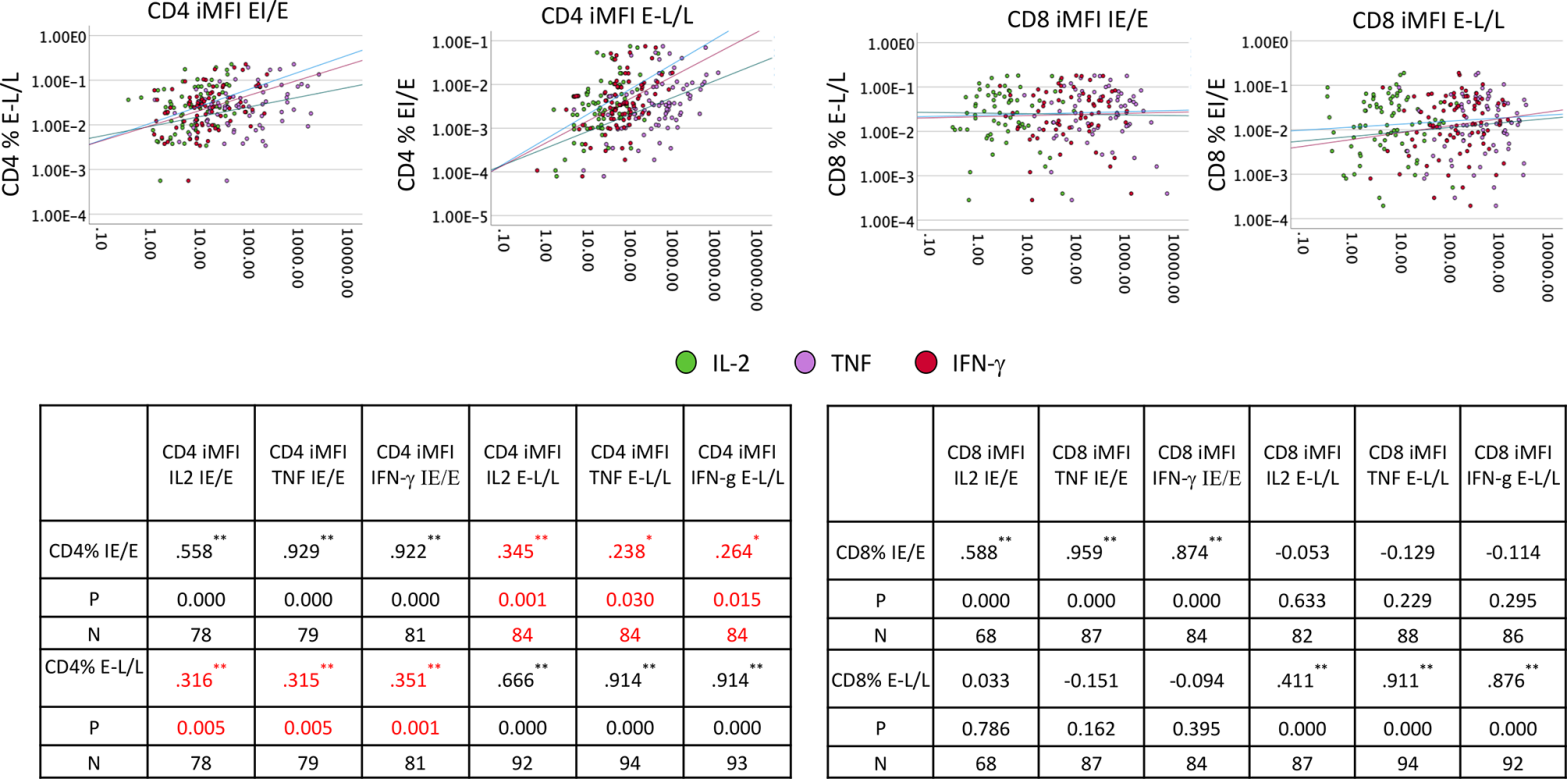
IE/E and E-L/L CD4 and CD8 T-cell response correlation. PBMCs from males (n=47) and females (n=47) were stimulated and processed for intracellular cytokine staining. Thereafter, the iMFI for each cytokine was calculated. Scatter plots show the association of IE/E protein specific with E-L/L protein-specific CD4 and CD8 T-cell responses. The inset tables show the Pearson R correlation coefficient for each association comparison with the level of significance. *p ≤ 0.05; **p ≤ 0.005.

We also investigated if there was an effect of the frequently reactivated responses to IE/E proteins on the response to any other proteins, potentially indicating that even incomplete reactivation would push all CMV-specific responses to a more differentiated phenotype. We, therefore, examined if the cytokine iMFIs of responses to IE/E (compared with E-L/L) proteins were associated with IE/E and E-L/L overall response sizes. For CD4 and CD8 T-cells we ran bivariate correlations between iMFI of IL-2, TNF, and IFN-γ responses to any CMV protein (14 pools) and the size of the responses directed against IE/E and E-L/L antigens (**Figure 6**). Note that correlations between the iMFI of IE/E protein-specific response with the overall size of the response to IE/E proteins would be expected as the iMFI as such incorporates the size of the response. However, a correlation of the iMFI of IE/E protein-specific responses and the size or iMFI of E-L/L protein specific responses would not be expected, unless reactivation (mostly marked by responses to IE/E protein-antigens) were to somehow enhance E-L/L-driven responses. Weak-to-moderate, yet significant, positive associations between the iMFIs (IL2, TNF and IFN-γ) of IE/E protein-specific CD4 T-cell responses and the size of the E-L/L protein specific CD4 T-cell response were found (**Figure 6**; see inset tables). This effect was not identified in CD8 T-cells, indicating that reactivation might have a stronger overall effect on the size of the CD4 T-cell response.

### Associations of Polyfunctionality With T-Cell Differentiation Do Not Differ Between Women and Men

**Figure 7:**
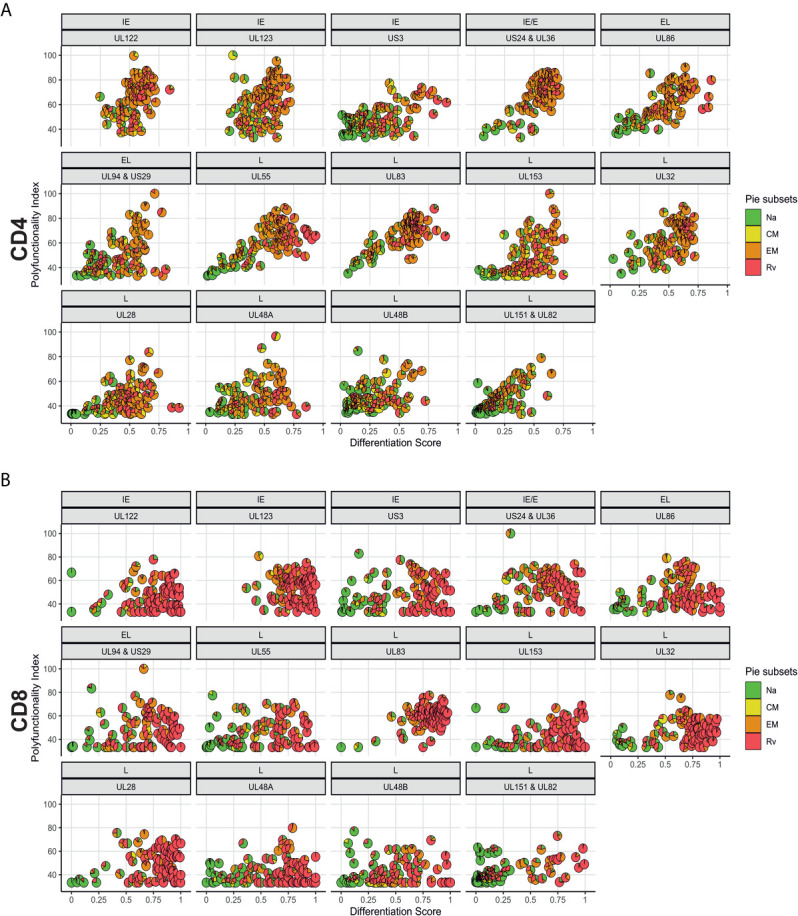
Polyfunctionality plotted *versus* differentiation score reveals distinct, protein-associated patterns. **(A)** CD4 and **(B)** CD8 T-cell polyfunctionality (Polyfunctionality Index) in relation to differentiation score for each CMV protein pool stratified according to the kinetics of the antigens. CMV protein designation and kinetic class are indicated at the top of each graph (in panels **A, B**). Pie charts show memory subset distributions including naive-like/stem-cell memory (Na, green), (CM, yellow), effector memory (EM, orange), and revertant memory (Rv, red).

In order to analyze associations of T-cell differentiation and polyfunctionality, we used the previously described polyfunctionality index (PI) (22), which allows one to summarize the composition of each T-cell response in terms of non-overlapping, functional Boolean subsets as a single number. The advantage of using this index is that polyfunctionality becomes a continuous variable that can be correlated with other parameters, for example, response size. We previously reported that, contrary to common belief, polyfunctionality is increased in large CMV-specific responses (26, 31). Here we sought to confirm this relationship and, in addition, explore differences between men and women in regard to protein-specific polyfunctionality, MFI/iMFI, memory differentiation and response size. For the purpose of representing memory differentiation in correlations, we generated a differentiation score (DSc). This score captures the percentages of CD4 and CD8 T-cells in each of the non-naïve compartments and assigns progressive weight to each compartment (see *Polyfunctionality Index and Differentiation Score* for a formal definition). The polyfunctionality of responses to CMV is more homogeneous (showing a tighter cluster of pies) for some CMV proteins (US3 and the four late proteins: UL28, UL48A, UL48B, UL151 & IL82) than others, in both CD4 and CD8 T-cells (**Figure 7**). Compared with CD4 T-cells, fewer CD8 T-cells produce IL-2, resulting in lower PI values for CD8 (compare **Figures 7A, B**). Also, the DSc is higher in CD8 than CD4 T-cells (with a DSc hardly exceeding 0.67) because CD4 T-cells rarely reacquire CD45RA expression (revertant cells) (**Figure 4**). Nevertheless, the correlation between polyfunctionality and differentiation score is less evident in CD8 than in CD4 T-cells, where there is a clear increase of polyfunctionality with higher differentiation (**Figure 7A**). Of note, the PI tends to rise along with the DSc until a certain point, after which it decreases, as most responses predominantly originate from revertant cells (Rv, red) (e.g., UL86, UL24&UL36, UL153 and UL48A/B in CD4 T-cells and UL55, UL28 and UL48A in CD8 T-cells, **Figure 7**). This agrees with our own previously published data comparing MFIs in different memory T-cell compartments (previously defined by CD27 and CD45RA) (26). While no significant sex differences were observed for CD4 and CD8 T-cell polyfunctionality to CMV, some non-significant trends towards lower polyfunctionality were apparent from UL153 in women (**Figure S8**).

### A Population View of CMV-Target Protein-Specific T-Cell Responses Reveals TNF-Dominated CD4 T-Cell Responses in Men That Are Absent in Women

**Figure 8:**
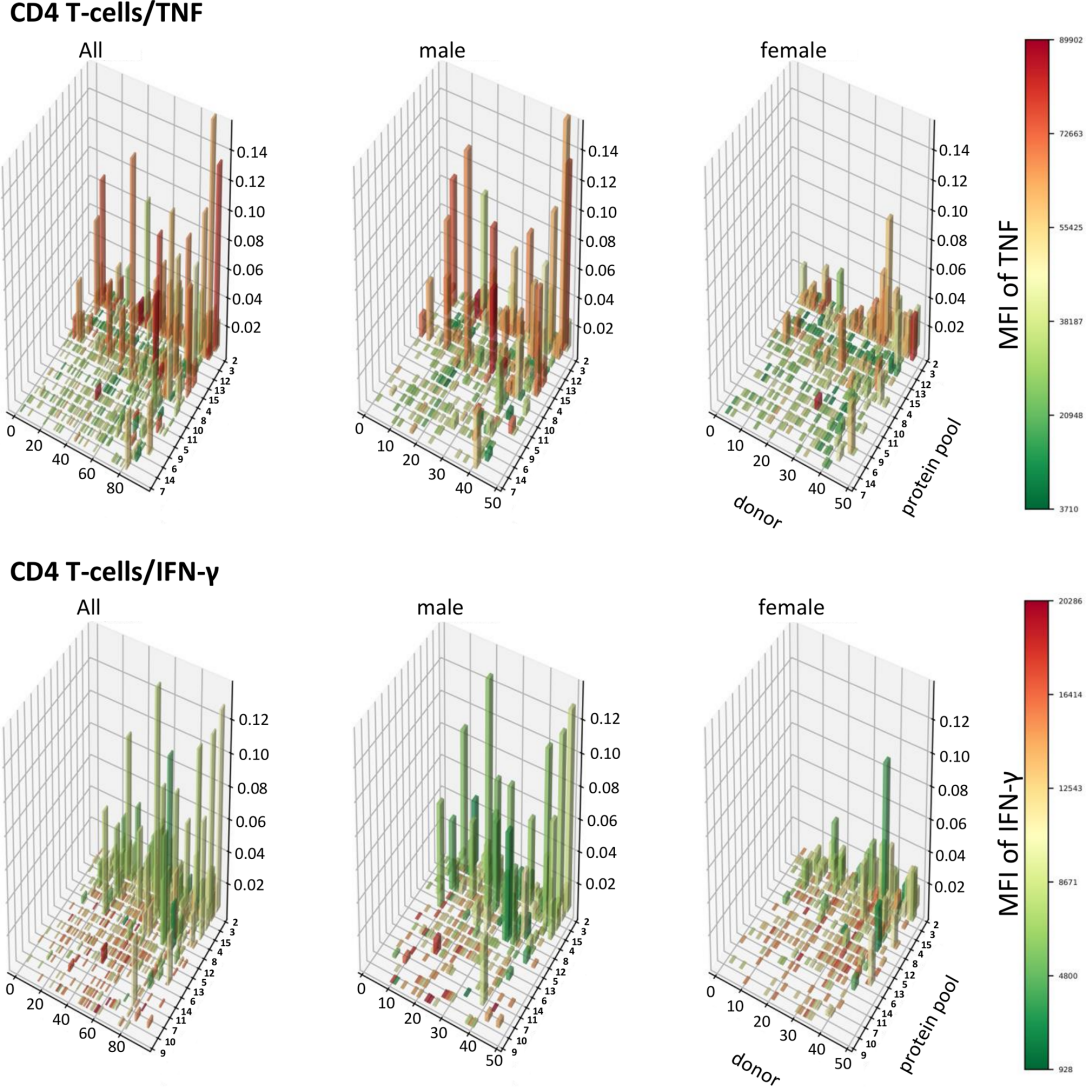
Large CD4 T-cell responses producing high levels of TNF are found preferentially in men. 3D bar charts show all individual CD4 T-cell responses to each CMV peptide pool in the entire population (left), men alone (middle) and women alone (right). Participants are ordered by increasing median T-cell response size from left to right with respect to the entire population. Peptide pools are ordered by increasing median response size induced from front to back, also with respect to the entire population. Bar height corresponds to the percentage of cytokine producing CD4 T-cells, i.e., response size. Bar color corresponds to the MFI of the measured cytokine according to the displayed scale.

**Figure 9:**
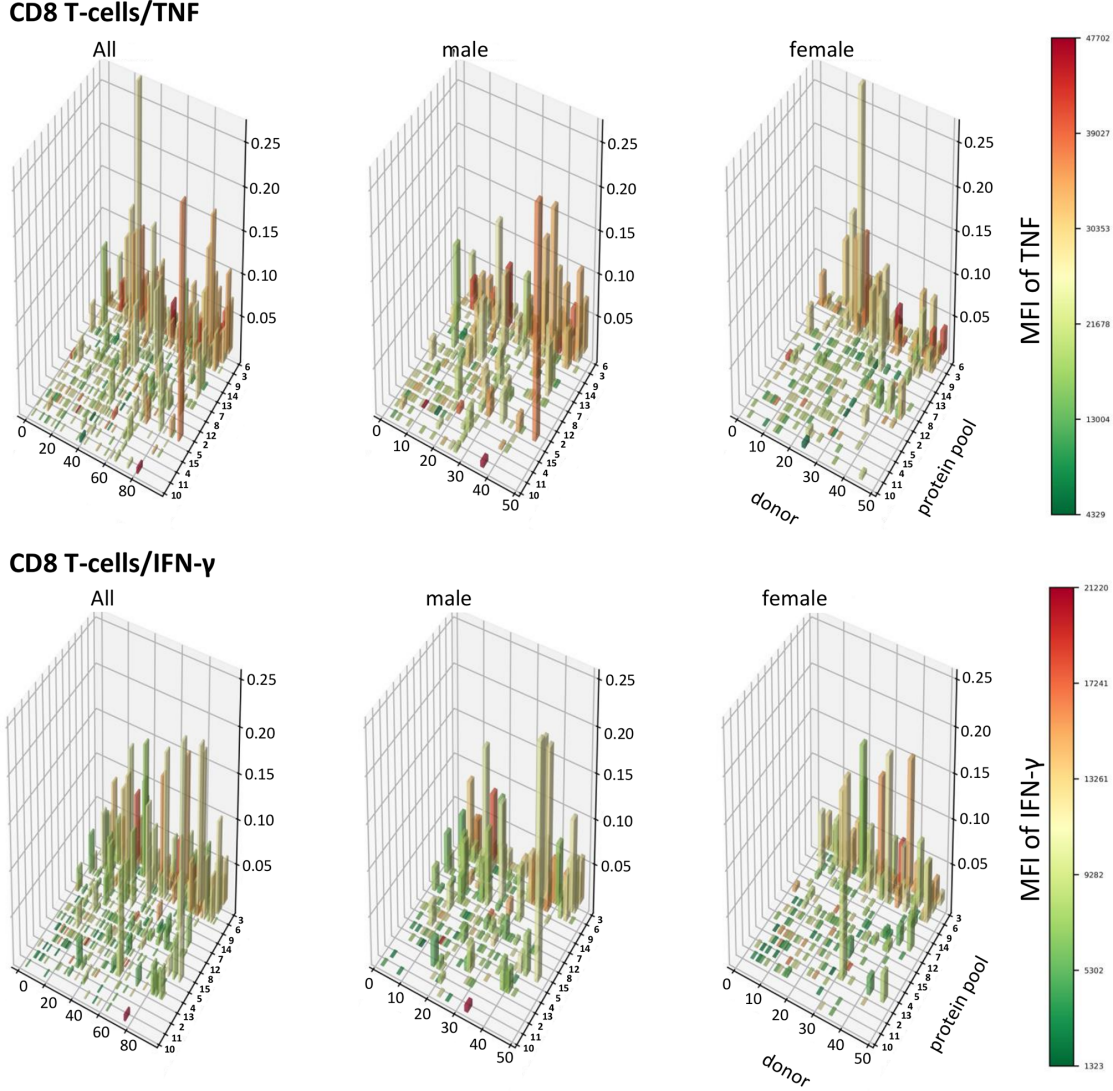
Patterns of TNF and IFN-γ producing CD8 T-cells appear similar in terms of size and distribution among men and women. 3D bar charts show all individual CD8 T-cell responses to each CMV peptide pool in the entire population (left), men alone (middle) and women alone (right). Participants are ordered by increasing median T-cell response size from left to right with respect to the entire population. Peptide pools are ordered by increasing median response size induced from front to back, also with respect to the entire population. Bar height corresponds to the percentage of cytokine producing CD8 T-cells, i.e., response size. Bar color corresponds to the MFI of the measured cytokine according to the displayed scale.

In order to visualize T-cell responses as a whole in the entire participant population, we plotted donor (x-axis) against protein pool (y-axis), response sizes in percentages of CD4 or CD8 T-cells (z-axis) as well as the MFI for TNF or IFN-γ (color gradient of the columns). This four-dimensional view shows a stark difference between women and men in regards to CD4 T-cells, in particular those producing TNF (**Figures 8**, **9**). It illustrates an excess of T-cell reactivity in terms of the measured cytokines in men and in particular the presence of response outliers. Sex differences in regards to IFN-γ producing CD4 T-cells were observed only in terms of response size. In CD8 T-cells sex differences were not nearly as obvious as in CD4 T-cells (**Figure 9**). The same analysis was performed using cells counts (cell/nL) obtaining similar results (**Figures S9**, **S10**).

In CD4 T-cells, response size measured in percentage (and cell counts) of TNF-producing cells was hence positively correlated with the MFI of TNF (i.e., the bigger response, the higher the MFI of TNF). However, response size measured in percentage (and cell counts) of IFN-γ-producing T-cells was negatively associated with the MFI of IFN-γ (the larger the response, the lower the MFI of IFN-γ). **Table 5** shows that in CD4 T-cells these positive and negative associations between response size and cytokine MFI were statistically significant for all dominant target proteins. Among CD8 T-cells the pattern was less clear overall, although clearly the MFIs of TNF and IFN-γ were significantly positively correlated with the respective response size in regards to several dominant responses but not the (most dominant) response to UL83. The pattern appeared less clear than in CD4 T-cells.

**Table 5:** Association of T-cell response size with cytokine MFI.

Response size in % of reference T-cell population	CD4 T-cells % IL-2 with MFI IL-2	CD4 T-cells % TNF with MFI TNF	CD4 T-cells % IFN-γ with MFI IFN-γ	CD8 T-cells % IL-2 with MFI IL-2	CD8 T-cells % TNF with MFI TNF	CD8 T-cells % IFN-γ with MFI IFN-γ
UL55^5^	0.028	0.358**	-0.310*	0.834**	-0.007	-0.220
UL83 (‘pp65’)	0.085	0.483***	-0.285**	-0.168	0.111	0.161
UL86	0.006	0.577***	-0.335**	0.350	-0.035	0.283
UL122	0.029	0.763***	-0.297*	-0.089	0.572**	0.409*
UL123 (‘IE-1’)	-0.096	0.366**	-0.168	0.009	0.353**	0.250*
UL153	-0.273	0.393	-0.269	-0.059	0.363	0.218
UL32	-0.076	0.194	-0.152	0.59*2	0.227	0.278
UL28	0.307	-0.218	-0.320	0.137	0.374**	0.360*
UL48A^a^	0.242	0.183	0.014	0.062	0.520*	0.977***
UL48B^a^	0.424*	0.007	-0.228	0.846**	0.432	0.253
US3	-0.153	0.533***	-0.357*	-0.311	0.036	-0.268
UL151& UL82	-0.049	-0.072	-0.199	0.456*	0.516***	0.061
UL94 & US29	-0.131	0.075	0.172	-0.151	-0.025	0.159
US24 & UL36	0.006	0.482***	-0.227	-0.068	0.519**	0.075
**Response size in cells/nL of reference T-cell population**	**CD4 T-cells cells/nL IL-2 with MFI IL-2**	**CD4 T-cells cells/nL TNF with MFI TNF**	**CD4 T-cells cells/nL IFN-γ with MFI IFN- γ**	**CD8 T-cells cells/nL IL-2 with MFI IL-2**	**CD8 T-cells cells/nL TNF with MFI TNF**	**CD8 T-cells cells/nL IFN- γ with MFI IFN- γ**
UL55^5^	0.048	0.264*	-0.337**	0.912**	0.007	-0.214
UL83 (‘pp65’)	0.175	0.501***	-0.245*	-0.100	0.213	0.190
UL86	0.055	0.592***	-0.331*	0.346	0.029	0.029
UL122	0.101	0.714***	-0.249	-0.173	0.635***	0.443*
UL123 (‘IE-1’)	-0.045	0.298*	-0.145	0.037	0.266	0.114
UL153	-0.316	0.394	-0.271	-0.167	0.287	0.187
UL32	-0.074	0.113	-0.102	0.038	0.284	0.211
UL28	0.447	-0.123	-0.258	0.098	0.303*	0.261
UL48A^a^	0.052	0.214	0.037	0.099	0.248	0.841*
UL48B^a^	0.312	0.010	-0.089	0.918**	0.560	0.351
US3	-0.106	0.636***	-0.362	-0.288	0.069	-0.216
UL151& UL82	0.037	0.060	-0.042	0.024	0.600***	0.034
UL94 & US29	-0.115	0.004	0.452*	-0.244	-0.086	0.121
US24 & UL36	0.024	0.387**	-0.197	-0.022	0.568**	0.059

a A panel of 19 CMV protein-spanning peptide pools was previously shown to correlate highly with the CD4 and CD8 T-cell response against 203 tested CMV proteins (19). The original panel contained UL99, UL103, and US32 in addition, but were left out here since responses were absent in >100 White British people. UL48 was divided into two pools (UL48A and UL48B), however, results were combined with respect to determining T-cell reactivity. *p ≤ 0.05, **p ≤ 0.01, ***p ≤ 0.001.

Statistical significances (p-values ≤0.05) are shown in bold.

## Discussion

Previous studies exploring the effect of biological sex on CMV-specific immunity had shown that CMV infection affects T-cell differentiation differently in women and men (3, 15). However, these studies did not explore the actual CMV-specific T-cell response in much detail, let alone the response to individual CMV proteins representing different phases of reactivation. The present study is the first to provide a detailed analysis of sex differences in the T-cell response to a range of dominant CMV T-cell target proteins.

Based on a selection of CMV proteins known to represent frequent and dominant T-cell response targets (19), we initially explored protein recognition (i.e., presence or absence of a response in regards to each protein in each individual) in a recently recruited cohort of 94 CMV-seropositive individuals. As expected, hierarchies in terms of protein recognition frequency differed between CD4 and CD8 T-cells. However, our results confirmed the previously identified hierarchy in an equally White British cohort with respect to the most dominant target proteins (26, 28). Minor differences detected in target protein response hierarchy between the historic cohort (19) and the present one may be related to differences in HLA-allele frequencies, since the historic cohort was ethnically more diverse.

Interestingly, the numbers of protein targets recognized by CD4 T-cells and CD8 T-cells in each individual were significantly correlated, despite the fact that CD4 and CD8 T-cells recognize peptides in the context of class-II and class-I MHC, respectively. This might be explained by an accumulation of HLA-alleles favoring CMV-peptide recognition during evolution. The distribution of T-cell responses in terms of recognized target proteins was very similar in women and men.

In agreement with the literature and our own previous reports (19, 20, 26, 28, 31), both the CD4 and CD8 T-cell compartments make a significant contribution to the CMV-specific T-cell response. CD4 T-cells may respond to antigens presented in the context of class-II MHC on APCs, but also endothelial cells. In addition, infected endothelial cells (and possibly other tissues) produce non-infectious exosomes containing CMV proteins. These are taken up by APCs and presented to CD4 T-cells (32). CD8 T-cells however seem to be mainly primed by cross presentation as CMV induces downmodulation of MHC-I molecules in infected cells (33). It was previously reported that immunogenic ORFs were represented across all kinetic classes of HCMV proteins, and it was also shown that the frequency of T-cells specific to a given protein was proportional to the representation of the corresponding ORF in the entire CMV proteome (19).

In this study, T-cell activation was identified by intracellular IL-2, TNF, and IFN-γ. This may be a limited panel, but these cytokines are critical for T-cell proliferation and effector function. TNF was the cytokine most frequently produced by activated CD4 T-cells, whereas among CD8 T-cells TNF and IFN-γ were equally dominant. We previously reported that the largest age-associated increase in CMV-specific T-cell responses occurred in regards to TNF-producing CD4 T-cells (28), which is of interest in this context, since the participants of this study were 60 years old or older. Our previous study, however, did not explore sex differences, but our current work clearly shows that men have overall larger T-cell responses to CMV proteins than women with a more pro-inflammatory CD4 T-cell component.

The combination of the three cytokines further allowed the division of activated T-cells into seven non-overlapping (Boolean) functional subsets. The relative contribution of these subsets to the overall response, as expected, differed a lot between CD4 and CD8 T-cells, and this was clearly the effect of CD8 T-cells not producing as much IL-2 as CD4 T-cells. The most ‘polyfunctional’ subset simultaneously producing IL-2, TNF, and IFN-γ was, therefore, much smaller in the CD8 T-cell compartment. In both T-cell compartments, however, the largest subset was generally subset 5 producing TNF and IFN-γ, but not IL-2. We found no sex difference in regards to the distribution of these subsets.

Another way of subdividing activated T-cell subsets is by their expression of markers defining ‘canonical’ memory T-cell subsets (i.e., naïve/naive-like stem cell memory, central memory, effector memory and revertant). This allows some conclusions as to the role that each protein plays in terms of driving T-cell memory. We analyzed the distribution of these subsets in regards to CD4 and CD8 responses to every single protein and also in terms of kinetic classes. The division of proteins by their assigned kinetic classes accounts for the fact that different CMV proteins may have more or less influence on T-cell responsiveness, depending on their presence or absence during the (frequent) reactivation of latent CMV infection (21, 27). IE protein expression will dominate immediately after reactivation and, in particular, if reactivation is abrogated and remains incomplete. The major immediate early promoter (MIEP) regulates IE protein expression and appears to be regulated itself by cellular chromatin activity, an increase of which is the common endpoint of a host of signaling pathways, particularly in inflammation (34). Memory subset composition was hence analyzed across all CMV proteins but also grouped by kinetic class, combining IE and E protein-specific responses in one group and E-L/L protein specific responses in the other. Responses to individual proteins varied significantly in terms of memory subset distribution both among CD4 and CD8 T-cells. Whereas the (normalized) composition of responses in terms of memory subset contribution indicated that there were smaller naïve and/or CM contributions to the CD4 and CD8 T-cell response in men, comparing the summed responses to these subsets showed that, not only the entire response (all memory subsets together) was bigger in men, but that in particular the EM and/or revertant subset contributions to responses against all CMV proteins and IE/E proteins were significantly bigger in men than in women. So, not only did men have bigger responses than women, but also the EM and Rv component of these responses appeared to contribute most to that difference.

It is a limitation of our work that viral load was not determined in our cohort. Previous work has shown that in otherwise healthy older people viral load is generally negative, however, digital droplet PCR may detect viral load in healthy older people over 70 years of age (35). We are unable to link T-cell responsiveness to viral loads since performing this very costly assay in all participants was outside the scope of our work. However, it is a possibility that a higher frequency of (potentially incomplete) CMV reactivation in men might contribute to a dominance of T-cell responsiveness to IE/E proteins. This would need to be addressed in future studies. The possible role of latency-associated proteins was not explored. Responses to these proteins appear to have very different functional characteristics than responses to lytic phase proteins (36), interestingly, however, changes to chromatin structure induced by histone deacetylases (HDACs) may lead to the transient expression of IE proteins without leading to full viral reactivation. This can be induced pharmacologically (37), but may also occur as a result of chromatin activity during cellular activation (34). It might be speculated that such an effect is more prominent in men.

Recent work suggests that persistent stimulation of CMV-specific T-cells may additionally result from a continuous low-level gene expression during latency with increasing evidence coming from both human and mouse models (38–40). This low-level gene expression may be involved in regulating the switch between latency and reactivation, and in the murine infection model appears to have random (stochastic) characteristics with a skewing to IE transcripts at about 8 months after latent infection of lungs (40). We speculate that increased proinflammatory cytokine secretion in response to immune stimulation in men (41) may increase low-level latent CMV gene expression and so contribute to the gender difference we have observed in the present study.

T-cell polyfunctionality is thought to be related to T-cell response efficacy after vaccination (42, 43). We used the polyfunctionality index (22) to gauge T-cell polyfunctionality as a compound measure allowing us to correlate it to other variables. Our data confirms that the most differentiated subsets (Rv) have reduced polyfunctionality, but the bulk of expanded and highly differentiated (EM and Rv) CD4 T-cells appears to retain two or more functions. This is particularly visible in the diagrams showing the differentiation score *versus* the polyfunctionality index. It is also in agreement with our previous report that expanded T-cells in older ages retain polyfunctionality (26).

The combination of polyfunctionality index and differentiation score observed in T-cells responding to different CMV proteins might reflect the role of these proteins in driving T-cell responsiveness. Responses to some proteins showed a wider spread along the axes than others. CD4 and CD8 T-cells responding to the UL123 protein, for example, generally had a high differentiation score and appeared more homogeneous in that sense than the response to US3, which included many cells at an early stage of memory differentiation. The T-cell response to UL83, one of the most dominant T-cell target proteins for both CD4 and CD8 T-cells, also included predominantly cells in an EM/Rv differentiation stage.

More inflammation, therefore, means more CMV reactivation. US3 is an IE protein and appears to be the earliest protein expressed that contributes to immune evasion. It does so by reducing the number of mature MHC complexes in the surface of infected cells (44, 45). Strong T-cell immunity against US3 might curtail this mechanism of immune evasion. US3 was recognized in about 50% of individuals with respect to CD4 T-cells (23 women and 22 men) and in about 40% with respect to CD8 T-cells (21 men and 15 women). The response to US3 was four to five times bigger in men than in women, but this difference reached statistical significance only in regards to IL-2+ CD4 T-cells. Differences with respect to the UL122 (IE-2) and UL123 (IE-1), two other dominant IE proteins, were not statistically significant, however there was a trend to larger TNF and IFN-γ UL123-specific CD8 T-cell responses in males. Overall, men appeared to have bigger responses to IE and E proteins than women. Responses to these immediate-early/early gene products may be of particular importance in driving the size of the T-cell immune response to CMV (44).

We also analyzed sex differences regarding the pro-inflammatory cytokine content of CMV-specific T-cell responses. Since we had not directly measured cytokine production in the supernatant of stimulated cells, we used the integrated MFI (iMFI) as a surrogate marker for the amount of cytokine produced (29, 46). With respect to CD4 and CD8 T-cells and all CMV proteins, the summated iMFI for TNF and IFN-γ was found higher in men than in women. The biggest sex differences in the CD4 T-cell compartment were related to IE/E proteins and in the CD8 compartment to E-L/L proteins. A limitation of these results might be that IE proteins are clearly overrepresented in our selection. This selection, however, was not random, but based on an analysis of overall protein immunodominance (19).

CD3/TCR downregulation occurs following T-cell recognition of MHC-peptide complexes (47) and, generally, correlates with T-cell activation and, more specifically, the strength of the initial activation (48, 49). The extent of CD3 downmodulation varied across the functional subsets defined by the presence or absence of IL-2, TNF, and IFN-γ. Sub1 (IL-2+/TNF+/IFN-γ+) and Sub5 (IL-2-/TNF+/IFN-γ+) showed the highest relative CD3 downregulation in CD4 T cells and Sub5 in CD8 T-cells. One benefit of CD3 downmodulation might be that, as a result of stronger downmodulation, high avidity, highly pro-inflammatory T-cell clones may compete less with lower avidity clones and so avoid excessive inflammatory responses (50). This would fit, for example, with the lesser CD3 downmodulation in men in regards to CD4 T-cell responses to a portion of the very abundant late protein UL48 (UL48A peptide pool) and would be commensurate with the equally observed trend to higher TNF-production by CD4 T-cells in response to the same peptide pool in men. However, our understanding of the biological significance of CD4 and CD8 T-cell responses to individual CMV proteins remains limited and we are unable to explain the majority of patterns of cytokine production *versus* CD3 downmodulation that we have observed. One might also speculate that effector T-cells that readily downregulate CD3/TCR may not progress towards terminal differentiation. This would favor the retention of IL-2 and TNF production, possibly alongside IFN-γ, and promote the emergence of long-lived polyfunctional T-cells with high avidity. The exact mechanisms of CD3/TCR downmodulation are still being debated. Beyond the TCR signal, they are involved in costimulation/immunoregulatory pathways [e.g., PD-1:PDL-1 (51)].

## Conclusions

Our work shows a number of statistically significant sex differences with respect to CMV-specific T-cell immunity (besides a number of statistically not significant, yet obvious trends). While CMV infection has been reported to cause broad changes to the peripheral blood B- and T-cell compartments with significant differences between women and men, sex differences regarding CMV protein-specific T-cells have not been widely discussed. The trends we observed all point in the same direction, suggesting that older men have a stronger and more inflammatory response to CMV than older women. This may be driven by more frequent CMV reactivation (or partial CMV reactivation) throughout life, which is suggested by higher T-cell responses to IE/E proteins in men. These findings are particularly relevant as CMV infection has been increasingly associated with CVD, which also has a higher incidence in men. Many of the differences we observed as trends would have reached statistical significance, had we not applied stringent multiple end-point corrections. While these remain a useful statistical safeguard against falling for random observations, future studies are warranted revisiting these observations. We are hoping that our work described here will have a seminal effect and make others examine gender differences in the adaptive immune system as we move towards a more personalized and stratified medicine.

## Data Availability Statement

The original contributions presented in the study are included in the article/**Supplementary Material**. Further inquiries can be directed to the corresponding authors.

## Ethics Statement

The studies involving human participants were reviewed and approved by UK National Research Ethics Service (NRES) London Centre (Reference 13/LO/1270). The patients/participants provided their written informed consent to participate in this study.

## Author Contributions

Conceptualization, FK. Methodology, GM and AB, and AP. Formal analysis, FK, BR, SC, ML, and AP. HLA typing and analysis, MH. Donor selection, CR. Writing—original draft preparation, FK, AP, and SC. Writing—review and editing, AB, GM, BR, SC, ML, FK, and AP. Project administration, AP and FK. Funding acquisition, CR and FK. All authors contributed to the article and approved the submitted version.

## Funding

This work was supported by The Dunhill Medical Trust, grant number R278/0213. AP is receiving funding from Miguel Servet CP19/00008, Instituto de Salud Carlos III & ERDF/ESF.

## Conflict of Interest

FK is employed part-time by JPT Peptide Technologies (Germany). ML is the proprietary owner of Funky Cells software.

The remaining authors declare that the research was conducted in the absence of any commercial or financial relationships that could be construed as a potential conflict of interest.

## Publisher’s Note

All claims expressed in this article are solely those of the authors and do not necessarily represent those of their affiliated organizations, or those of the publisher, the editors and the reviewers. Any product that may be evaluated in this article, or claim that may be made by its manufacturer, is not guaranteed or endorsed by the publisher.

## References

[1] Solana R Tarazona R Aiello AE Akbar AN Appay V Beswick M. CMV and Immunosenescence: From Basics to Clinics. Immun Ageing (2012) 9(1):23. 10.1186/1742-4933-9-23 23114110 PMC3585851

[2] Pourgheysari B Khan N Best D Bruton R Nayak L Moss PA. The Cytomegalovirus-Specific CD4+ T-Cell Response Expands With Age and Markedly Alters the CD4+ T-Cell Repertoire. J Virol (2007) 81(14):7759–65. 10.1128/JVI.01262-06 PMC1933343 17409149

[3] Di Benedetto S Derhovanessian E Steinhagen-Thiessen E Goldeck D Muller L Pawelec G. Impact of Age, Sex and CMV-Infection on Peripheral T Cell Phenotypes: Results From the Berlin BASE-II Study. Biogerontology (2015) 16(5):631–43. 10.1007/s10522-015-9563-2 25732234

[4] Campos C Pera A Sanchez-Correa B Alonso C Lopez-Fernandez I Morgado S. Effect of Age and CMV on NK Cell Subpopulations. Exp Gerontol (2014) 54:130–7. 10.1016/j.exger.2014.01.008 24440462

[5] Reed RG Al-Attar A Presnell SR Lutz CT Segerstrom SC. A Longitudinal Study of the Stability, Variability, and Interdependencies Among Late-Differentiated T and NK Cell Subsets in Older Adults. Exp Gerontol (2019) 121:46–54. 10.1016/j.exger.2019.03.006 30885717 PMC6482456

[6] van der Heiden M van Zelm MC Bartol SJW de Rond LGH Berbers GAM Boots AMH. Differential Effects of Cytomegalovirus Carriage on the Immune Phenotype of Middle-Aged Males and Females. Sci Rep (2016) 6:26892. 10.1038/srep26892 27243552 PMC4886678

[7] Vallejo AN Nestel AR Schirmer M Weyand CM Goronzy JJ. Aging-Related Deficiency of CD28 Expression in CD4+ T Cells Is Associated With the Loss of Gene-Specific Nuclear Factor Binding Activity. J Biol Chem (1998) 273(14):8119–29. 10.1074/jbc.273.14.8119 9525915

[8] Hooper M Kallas EG Coffin D Campbell D Evans TG Looney RJ. Cytomegalovirus Seropositivity Is Associated With the Expansion of CD4+CD28- and CD8+CD28- T Cells in Rheumatoid Arthritis. J Rheumatol (1999) 26(7):1452–7. 10405929

[9] Pawelec G Koch S Franceschi C Wikby A. Human Immunosenescence: Does It Have an Infectious Component? Ann NY Acad Sci (2006) 1067:56–65. 10.1196/annals.1354.009 16803971

[10] Pera A Caserta S Albanese F Blowers P Morrow G Terrazzini N. CD28^null^ Pro-Atherogenic CD4 T-Cells Explain the Link Between CMV Infection and an Increased Risk of Cardiovascular Death. Theranostics (2018) 8(16):4509–19. 10.7150/thno.27428 PMC6134924 30214635

[11] Cannon MJ Schmid DS Hyde TB. Review of Cytomegalovirus Seroprevalence and Demographic Characteristics Associated With Infection. Rev Med Virol (2010) 20(4):202–13. 10.1002/rmv.655 20564615

[12] Klenerman P Dunbar PR. CMV and the Art of Memory Maintenance. Immunity (2008) 29(4):520–2. 10.1016/j.immuni.2008.09.008 18957264

[13] Bots SH Peters SAE Woodward M. Sex Differences in Coronary Heart Disease and Stroke Mortality: A Global Assessment of the Effect of Ageing Between 1980 and 2010. BMJ Glob Health (2017) 2(2):e000298. 10.1136/bmjgh-2017-000298 PMC5435266 28589033

[14] Vrijenhoek JE Den Ruijter HM De Borst GJ de Kleijn DP De Vries JP Bots ML. Sex Is Associated With the Presence of Atherosclerotic Plaque Hemorrhage and Modifies the Relation Between Plaque Hemorrhage and Cardiovascular Outcome. Stroke (2013) 44(12):3318–23. 10.1161/STROKEAHA.113.002633 24130138

[15] Kirkham F Pera A Simanek AM Bano A Morrow G Reus B. Cytomegalovirus Infection Is Associated With an Increase in Aortic Stiffness in Older Men Which May Be Mediated in Part by CD4 Memory T-Cells. Theranostics (2021) 11(12):5728–41. 10.7150/thno.58356 PMC8058738 33897878

[16] Villacres MC Longmate J Auge C Diamond DJ. Predominant Type 1 CMV-Specific Memory T-Helper Response in Humans: Evidence for Gender Differences in Cytokine Secretion. Hum Immunol (2004) 65(5):476–85. 10.1016/j.humimm.2004.02.021 15172447

[17] Kern F Faulhaber N Frommel C Khatamzas E Prosch S Schonemann C. Analysis of CD8 T Cell Reactivity to Cytomegalovirus Using Protein-Spanning Pools of Overlapping Pentadecapeptides. Eur J Immunol (2000) 30(6):1676–82. 10.1002/1521-4141(200006)30:6<1676::AID-IMMU1676>3.0.CO;2-V 10898504

[18] Maecker HT Dunn HS Suni MA Khatamzas E Pitcher CJ Bunde T. Use of Overlapping Peptide Mixtures as Antigens for Cytokine Flow Cytometry. J Immunol Methods (2001) 255(1-2):27–40. 10.1016/s0022-1759(01)00416-1 11470284

[19] Sylwester AW Mitchell BL Edgar JB Taormina C Pelte C Ruchti F. Broadly Targeted Human Cytomegalovirus-Specific CD4+ and CD8+ T Cells Dominate the Memory Compartments of Exposed Subjects. J Exp Med (2005) 202(5):673–85. 10.1084/jem.20050882 PMC2212883 16147978

[20] Sylwester A Nambiar KZ Caserta S Klenerman P Picker LJ Kern F. A New Perspective of the Structural Complexity of HCMV-Specific T-Cell Responses. Mech Ageing Dev (2016) 158:14–22. 10.1016/j.mad.2016.03.002 26957355

[21] Wathen MW Stinski MF. Temporal Patterns of Human Cytomegalovirus Transcription: Mapping the Viral RNAs Synthesized at Immediate Early, Early, and Late Times After Infection. J Virol (1982) 41(2):462–77. 10.1128/JVI.41.2.462-477.1982 PMC256775 6281461

[22] Larsen M Sauce D Arnaud L Fastenackels S Appay V Gorochov G. Evaluating Cellular Polyfunctionality With a Novel Polyfunctionality Index. PloS One (2012) 7(7):e42403. 10.1371/journal.pone.0042403 22860124 PMC3408490

[23] R-Core-Team. R: A Language and Environment for Statistical Computing. Vienna, Austria: R Foundation for Statistical Computing (2019). Available at: https://www.R-project.org/.

[24] Wickham H. Ggplot2: Elegant Graphics for Data Analysis. New York: Springer-Verlag (2016).

[25] Yu G. Scatterpie: Scatter Pie Plot. R Package Version 0.1.5. (2020). Available at: https://CRAN.R-project.org/package=scatterpie.

[26] Bajwa M Vita S Vescovini R Larsen M Sansoni P Terrazzini N. Functional Diversity of Cytomegalovirus-Specific T Cells Is Maintained in Older People and Significantly Associated With Protein Specificity and Response Size. J Infect Dis (2016) 214(9):1430–7. 10.1093/infdis/jiw371 PMC5079367 27521364

[27] Chambers J Angulo A Amaratunga D Guo H Jiang Y Wan JS. DNA Microarrays of the Complex Human Cytomegalovirus Genome: Profiling Kinetic Class With Drug Sensitivity of Viral Gene Expression. J Virol (1999) 73(7):5757–66. 10.1128/JVI.73.7.5757-5766.1999 PMC112636 10364327

[28] Bajwa M Vita S Vescovini R Larsen M Sansoni P Terrazzini N. CMV-Specific T-Cell Responses at Older Ages: Broad Responses With a Large Central Memory Component May Be Key to Long-Term Survival. J Infect Dis (2017) 215(8):1212–20. 10.1093/infdis/jix080 PMC5854018 28199648

[29] Shooshtari P Fortuno ES 3rd Blimkie D Yu M Gupta A Kollmann TR. Correlation Analysis of Intracellular and Secreted Cytokines via the Generalized Integrated Mean Fluorescence Intensity. Cytometry A (2010) 77(9):873–80. 10.1002/cyto.a.20943 PMC2930075 20629196

[30] van den Berg SPH Pardieck IN Lanfermeijer J Sauce D Klenerman P van Baarle D. The Hallmarks of CMV-Specific CD8 T-Cell Differentiation. Med Microbiol Immunol (2019) 208(3-4):365–73. 10.1007/s00430-019-00608-7 PMC6647465 30989333

[31] Lachmann R Bajwa M Vita S Smith H Cheek E Akbar A. Polyfunctional T Cells Accumulate in Large Human Cytomegalovirus-Specific T Cell Responses. J Virol (2012) 86(2):1001–9. 10.1128/jvi.00873-11 PMC3255847 22072753

[32] Walker JD Maier CL Pober JS. Cytomegalovirus-Infected Human Endothelial Cells Can Stimulate Allogeneic CD4+ Memory T Cells by Releasing Antigenic Exosomes. J Immunol (2009) 182(3):1548–59. 10.4049/jimmunol.182.3.1548 PMC2630120 19155503

[33] Busche A Jirmo AC Welten SP Zischke J Noack J Constabel H. Priming of CD8+ T Cells Against Cytomegalovirus-Encoded Antigens Is Dominated by Cross-Presentation. J Immunol (2013) 190(6):2767–77. 10.4049/jimmunol.1200966 23390296

[34] Mason R Groves IJ Wills MR Sinclair JH Reeves MB. Human Cytomegalovirus Major Immediate Early Transcripts Arise Predominantly From the Canonical Major Immediate Early Promoter in Reactivating Progenitor-Derived Dendritic Cells. J Gen Virol (2020) 101(6):635–44. 10.1099/jgv.0.001419 PMC7414444 32375946

[35] Parry HM Zuo J Frumento G Mirajkar N Inman C Edwards E. Cytomegalovirus Viral Load Within Blood Increases Markedly in Healthy People Over the Age of 70 Years. Immun Ageing (2016) 13:1. 10.1186/s12979-015-0056-6 26734066 PMC4700608

[36] Mason GM Jackson S Okecha G Poole E Sissons JG Sinclair J. Human Cytomegalovirus Latency-Associated Proteins Elicit Immune-Suppressive IL-10 Producing CD4(+) T Cells. PloS Pathog (2013) 9(10):e1003635. 10.1371/journal.ppat.1003635 24130479 PMC3795018

[37] Krishna BA Lau B Jackson SE Wills MR Sinclair JH Poole E. Transient Activation of Human Cytomegalovirus Lytic Gene Expression During Latency Allows Cytotoxic T Cell Killing of Latently Infected Cells. Sci Rep (2016) 6:24674. 10.1038/srep24674 27091512 PMC4835774

[38] Shnayder M Nachshon A Krishna B Poole E Boshkov A Binyamin A. Defining the Transcriptional Landscape During Cytomegalovirus Latency With Single-Cell RNA Sequencing. mBio (2018) 9(2):e00013–18. 10.1128/mBio.00013-18 PMC5850328 29535194

[39] Cheng S Caviness K Buehler J Smithey M Nikolich-Zugich J Goodrum F. Transcriptome-Wide Characterization of Human Cytomegalovirus in Natural Infection and Experimental Latency. Proc Natl Acad Sci USA (2017) 114(49):E10586–95. 10.1073/pnas.1710522114 PMC5724264 29158406

[40] Griessl M Renzaho A Freitag K Seckert CK Reddehase MJ Lemmermann NAW. Stochastic Episodes of Latent Cytomegalovirus Transcription Drive CD8 T-Cell “Memory Inflation” and Avoid Immune Evasion. Front Immunol (2021) 12:668885. 10.3389/fimmu.2021.668885 33968074 PMC8100209

[41] Aulock SV Deininger S Draing C Gueinzius K Dehus O Hermann C. Gender Difference in Cytokine Secretion on Immune Stimulation With LPS and LTA. J Interferon Cytokine Res (2006) 26(12):887–92. 10.1089/jir.2006.26.887 17238831

[42] Precopio ML Betts MR Parrino J Price DA Gostick E Ambrozak DR. Immunization With Vaccinia Virus Induces Polyfunctional and Phenotypically Distinctive CD8(+) T Cell Responses. J Exp Med (2007) 204(6):1405–16. 10.1084/jem.20062363 PMC2118607 17535971

[43] Boyd A Almeida JR Darrah PA Sauce D Seder RA Appay V. Pathogen-Specific T Cell Polyfunctionality Is a Correlate of T Cell Efficacy and Immune Protection. PloS One (2015) 10(6):e0128714. 10.1371/journal.pone.0128714 26046523 PMC4457486

[44] Hegde NR Tomazin RA Wisner TW Dunn C Boname JM Lewinsohn DM. Inhibition of HLA-DR Assembly, Transport, and Loading by Human Cytomegalovirus Glycoprotein US3: A Novel Mechanism for Evading Major Histocompatibility Complex Class II Antigen Presentation. J Virol (2002) 76(21):10929–41. 10.1128/jvi.76.21.10929-10941.2002 PMC136637 12368336

[45] Liu Z Winkler M Biegalke B. Human Cytomegalovirus: Host Immune Modulation by the Viral US3 Gene. Int J Biochem Cell Biol (2009) 41(3):503–6. 10.1016/j.biocel.2008.10.012 18992841

[46] Darrah PA Patel DT De Luca PM Lindsay RW Davey DF Flynn BJ. Multifunctional TH1 Cells Define a Correlate of Vaccine-Mediated Protection Against Leishmania Major. Nat Med (2007) 13(7):843–50. 10.1038/nm1592 17558415

[47] Valitutti S Muller S Dessing M Lanzavecchia A. Different Responses are Elicited in Cytotoxic T Lymphocytes by Different Levels of T Cell Receptor Occupancy. J Exp Med (1996) 183(4):1917–21. 10.1084/jem.183.4.1917 PMC2192499 8666949

[48] Bachmann MF Oxenius A Speiser DE Mariathasan S Hengartner H Zinkernagel RM. Peptide-Induced T Cell Receptor Down-Regulation on Naive T Cells Predicts Agonist/Partial Agonist Properties and Strictly Correlates With T Cell Activation. Eur J Immunol (1997) 27(9):2195–203. 10.1002/eji.1830270912 9341759

[49] Viola A Lanzavecchia A. T Cell Activation Determined by T Cell Receptor Number and Tunable Thresholds. Science (1996) 273(5271):104–6. 10.1126/science.273.5271.104 8658175

[50] Gallegos AM Xiong H Leiner IM Susac B Glickman MS Pamer EG. Control of T Cell Antigen Reactivity *via* Programmed TCR Downregulation. Nat Immunol (2016) 17(4):379–86. 10.1038/ni.3386 PMC4803589 26901151

[51] Escors D Bricogne C Arce F Kochan G Karwacz K. On the Mechanism of T Cell Receptor Down-Modulation and Its Physiological Significance. J Biosci Med (2011) 1(1):2011.5. 22318485 PMC3272427

