# Supplementary material for: In-Depth Profiling of T-Cell Responsiveness to Commonly Recognized CMV Antigens in Older People Reveals Important Sex Differences
Source: Front Immunol. 2021 Aug 13;12:707830. doi: 10.3389/fimmu.2021.707830 (PMC8414256; doi:10.3389/fimmu.2021.707830)
Supplement: Supplementary file 1 [file DataSheet_1.pdf]

Supplementary Materials:

Materials and methods

CMV-peptides

Each pool included 25 µg per peptide (PepMixTM, JPT Peptide Technologies, Berlin, Germany) dissolved in 100 µL of DMSO (Sigma-Aldrich, Gillingham, UK) (0.25 µg/µL). T-cells were stimulated with 2 µL of the 0.25 µg/µL CMV-peptide solutions for a final concentration of 1 µg/mL per peptide. All assays included a positive control consisting of 1 µg/ml (final concentration) of Staphylococcal enterotoxin B from *Staphylococcus aureus* (SEB, Sigma) dissolved in DMSO, and a negative control consisting of 2 µL of DMSO alone (i.e., the peptide solvent). The list of all CMV proteins used in the study is provided in Table 1.

CMV-reactivity of T-cells

CMV-specific T-cells were activated by incubating PBMCs for 16 h overnight (37 °C, humidified 5% CO_2 atmosphere) with peptide pools, positive and negative controls. Per tube $1x10^6$ PBMCs were incubated with 2 µL of the corresponding CMV peptide pool, 1 µL of SEB solution (positive control, final concentration 1 µg/mL) or 2 µL of DMSO (negative control) in complete RPMI media (final incubation volume 250 µL). After 2 h in a standard incubator (37°C, humidified 5% CO2 atmosphere) 250 µL of complete RPMI containing 10 µg/mL of Brefeldin A (BFA) was added to each tube, resulting in a final volume of 500 µL for the remaining incubation time (14 h). Following, each tube was incubated for 10 min at 37 °C (water bath) with 100 µL of 20 mM EDTA (Sigma). Subsequently, samples were washed with 3 mL of wash buffer (PBS with 0.5% w/v BSA and 0.1% w/v sodium azide, (Sigma) and centrifuged at 400*g* for 8 min at RT ('wash step'). Samples were then surface-stained (30 min at 4 °C in the dark) with fluorochrome conjugated monoclonal antibodies including anti-CD3 Pacific Blue, anti-CD4 BV510, anti-CD8a PE-Cy7, anti-CCR7 BV605, and anti-CD45RA PerCP/Cy5.5 (all Biolegend), followed by a wash step. Following the surface stain 1ml of 1x BD 'FACS Lysing Solution' (BD) was added to each tube and tubes incubated for 10 min in the dark. Another wash step followed and subsequently, BD 'Permeabilization solution 2' was added to each tube and samples were incubated for 10 min in the dark. After a further wash step, cells were stained intracellularly with anti-IL-2 APC, anti-TNFα AF 700 and anti-IFN-γ FITC (all Biolegend) for 30 min at 4 °C in the dark followed by a wash step. Then, 1 mL of

0.5% paraformaldehyde (in water) was added to each tube and incubated in the dark for 5 min. After a final wash step cells were stored at 4 °C in the dark until acquisition.

Data acquisition and analysis

Following staining, samples were acquired on an LSR II flow-cytometer (BD) and analyzed with FlowJo v9.x software (Tree Star Inc., Ashland, OR). Gating strategy is described in Figure S1.

Quality control for flow-cytometry

Comparable day-to-day performance of the LSR II flow-cytometer was ascertained by running CS&T calibration beads (BD) on a daily basis. In addition, 8-peak Rainbow beads were used prior to every run in order to adjust PMTs in such a way that with respect to each detector, every peak was always in the same channel.

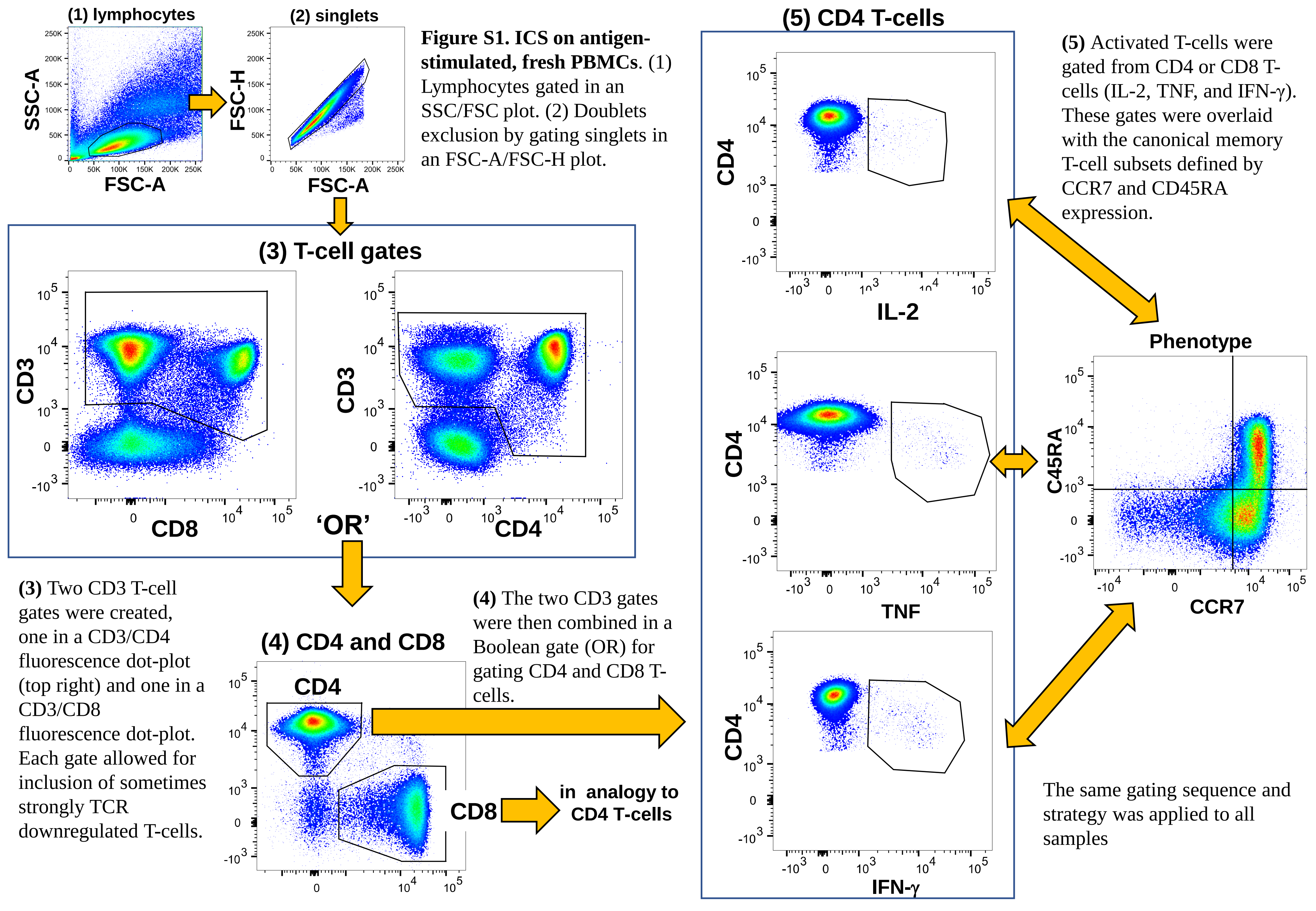

Figure S1. ICS on antigen-stimulated, fresh PBMCs. (1) Lymphocytes gated in an SSC/FSC plot. (2) Doublets exclusion by gating singlets in an FSC-A/FSC-H plot.

(3) Two CD3 T-cell gates were created, one in a CD3/CD4 fluorescence dot-plot (top right) and one in a CD3/CD8 fluorescence dot-plot. Each gate allowed for inclusion of sometimes strongly TCR downregulated T-cells.

(4) The two CD3 gates were then combined in a Boolean gate (OR) for gating CD4 and CD8 T-cells.

(5) Activated T-cells were gated from CD4 or CD8 T-cells (IL-2, TNF, and IFN-γ). These gates were overlaid with the canonical memory T-cell subsets defined by CCR7 and CD45RA expression.

The same gating sequence and strategy was applied to all samples

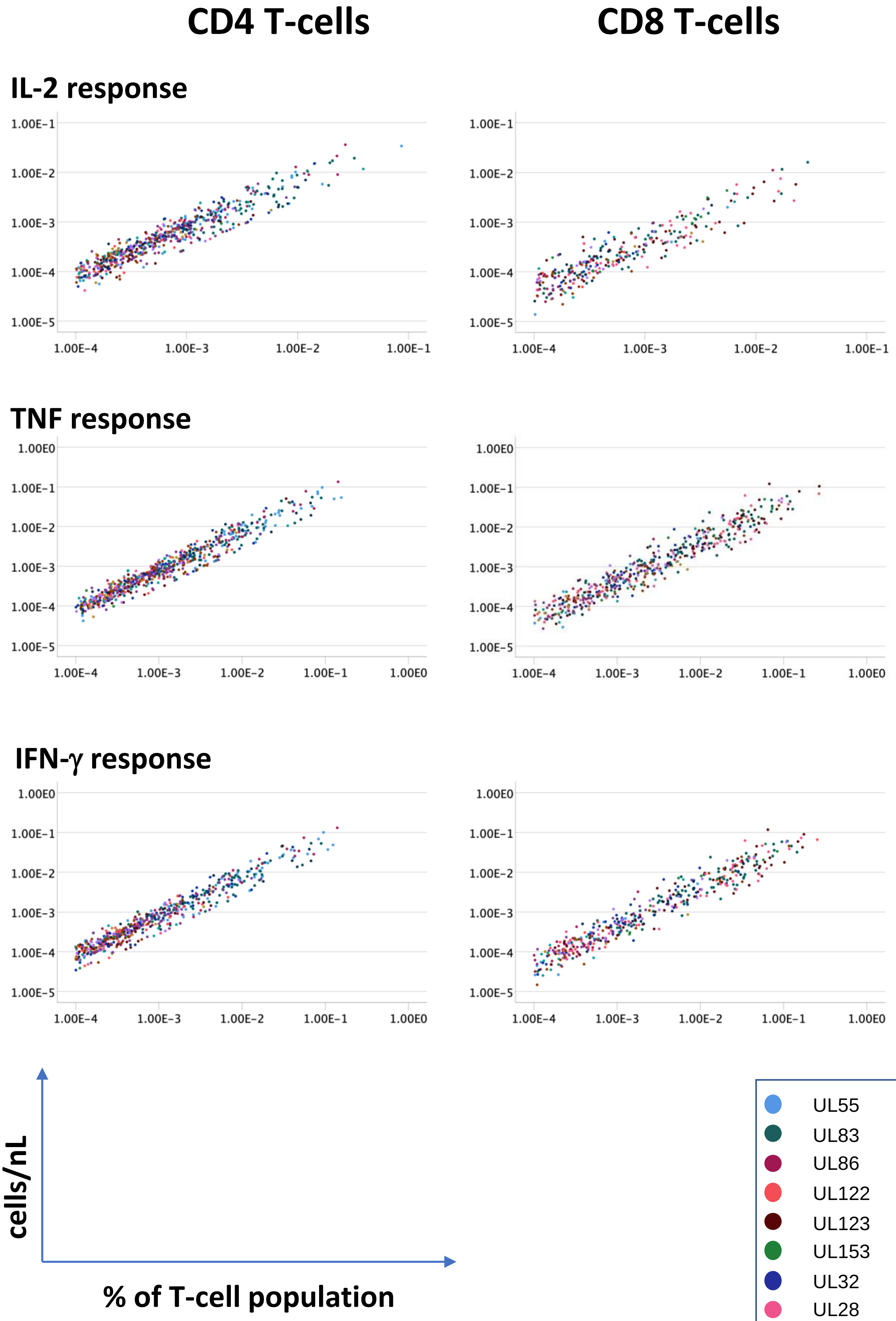

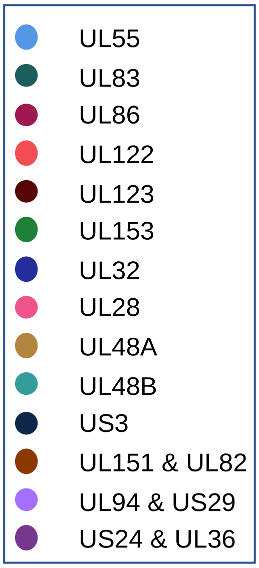

Figure S2. Correlation between counts (cells per nL of blood, y-axis) and percentage (y-axis) of T-cells responding to each CMV protein pool.

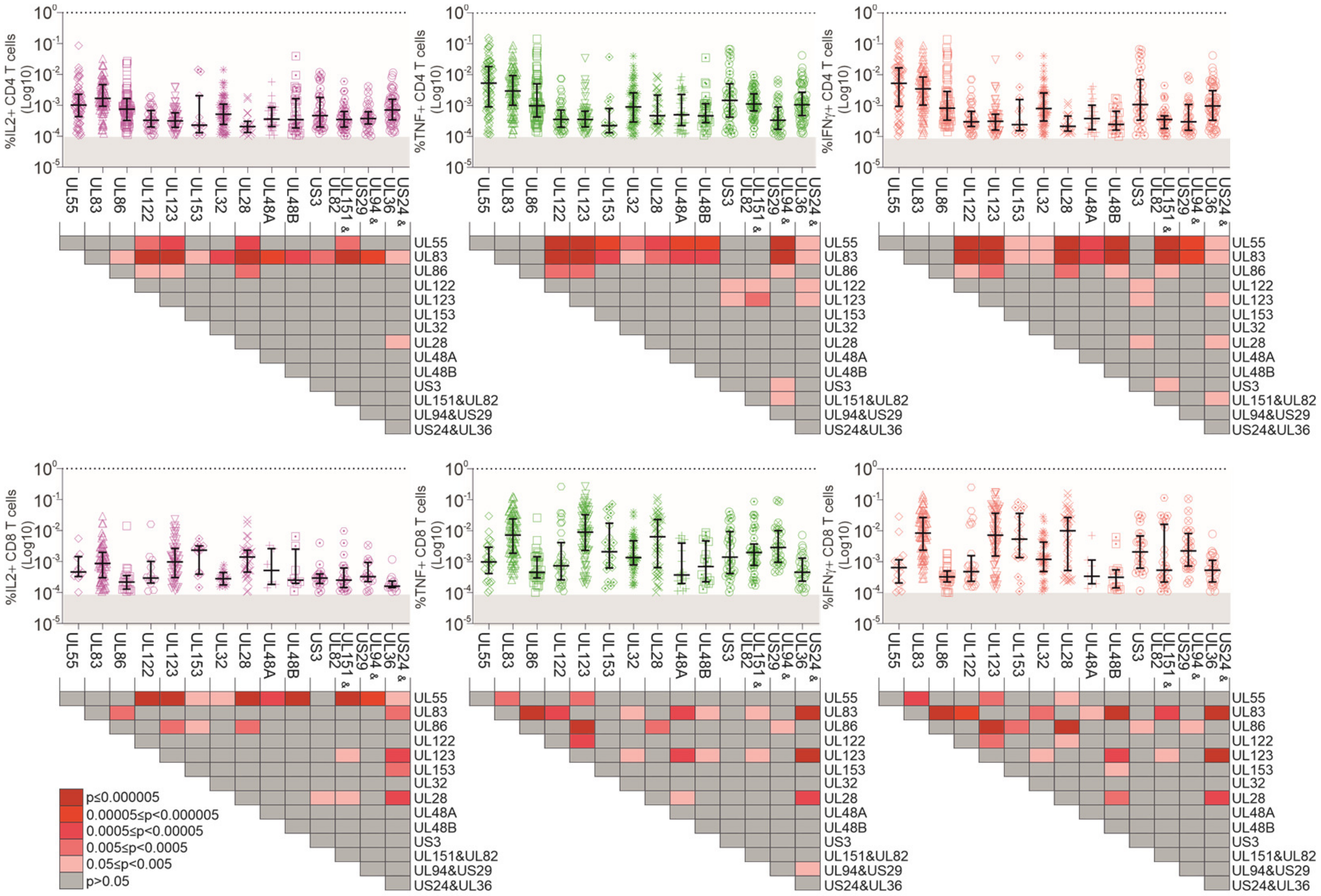

Figure S3. CD4 and CD8 T-cell reactivity against 14 different CMV peptide pools representing 16 CMV proteins. For each individual of the cohort (n=94), fresh PBMCs were stimulated with specific CMV protein peptide mixes (14 peptide mixes). CMV protein-specific cytokine (IL-2, TNF and IFN-γ) responses were analyzed after exclusion of responses below the 1/10,000 threshold (grey shaded area of each plot), in gated CD4 and CD8 T-cells (upper and lower panels, respectively). Scatter plots show the frequencies of CD4 and CD8 T-cells responding to the indicated CMV peptide pools (as highlighted by different empty symbols). Error bars show median and interquartile range. Statistical differences in response sizes between different CMV proteins were assessed in Kruskal-Wallis Tests (p<0.000001, for all cytokines in CD4 and CD8 T-cells) (non-parametric statistical comparisons), followed by Dunn's multiple comparison correction to assess adjusted *p*-values. These are graphically depicted at the bottom of triangularized matrices. Within a triangular matrix, each row represents a CMV protein pool scored against any other protein pool used in the study (columns of the triangularized matrix) for statistical significance (grey shaded cells: $p>0.05$, non-significant and then progressively darker shades of red for $0.05 \leq p < 0.005$, $0.005 \leq p < 0.0005$, $0.0005 \leq p < 0.00005$, $0.00005 \leq p < 0.000005$ and $p \leq 0.000005$ to indicate five levels of significance, respectively).

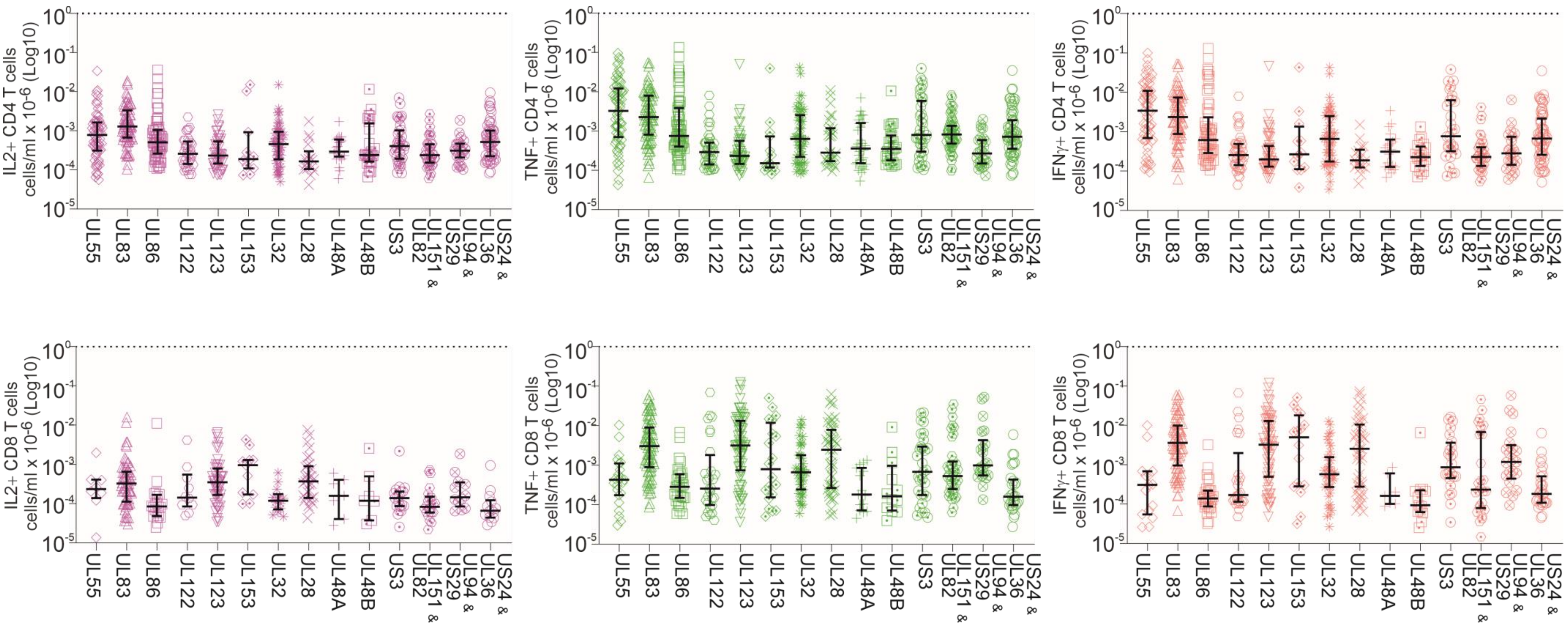

Figure S4. CD4 and CD8 T-cell reactivity against 14 different CMV peptide pools representing 16 CMV proteins. For each individual of the cohort (n=94), fresh PBMCs were stimulated with specific CMV protein peptide mixes (14 peptide mixes). CMV protein-specific cytokine (IL-2, TNF and IFN-γ) responses were analyzed after exclusion of responses below the 1/10,000 threshold in gated CD4 and CD8 T-cells (upper and lower panels, respectively). Scatter plots show cell counts/nL of CD4 and CD8 T-cells responding to the indicated CMV peptide pools (as highlighted by different empty symbols). Error bars show median and interquartile range.

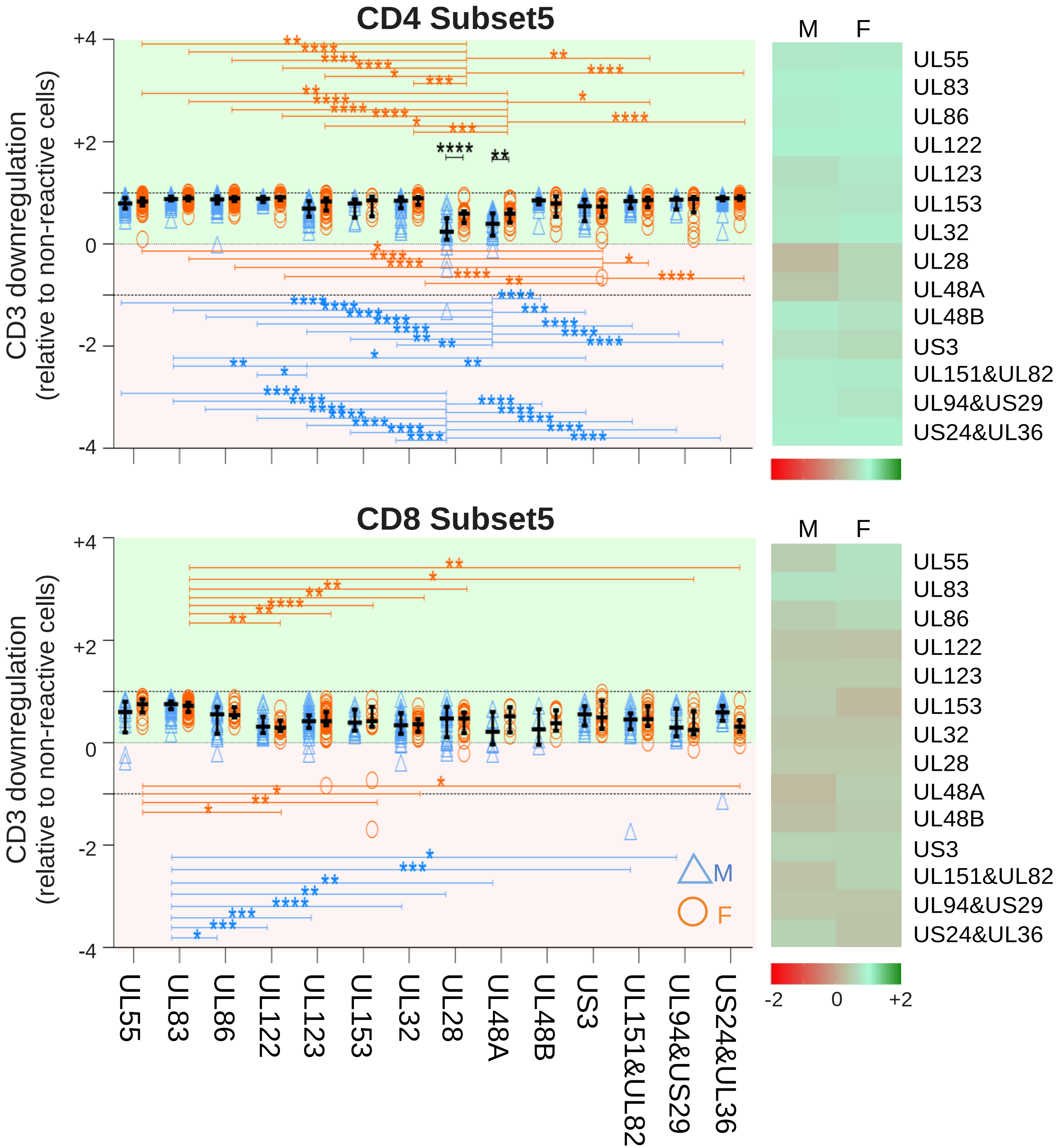

Figure S5. CD3 downregulation in CD4 T-cell Subset 5 differs between men and women. PBMCs from men (n=47) and females (n=47) were stimulated with specific CMV-protein peptide mixes and processed for intracellular cytokine staining, excluding responses below the 1/10,000 threshold. Levels of CD3 downregulation were calculated relative to the MFI of the non-responsive T-cell population. Heatmaps show CD3 downmodulation in males (M) compared to females (F). Darker shades of red represent low and darker shades of green high CD3 downmodulation. Scatter plots show CD3 downregulation in males (blue triangles) and females (orange circles). Error bars show median and interquartile range. Statistically significant differences (i) between sexes are indicated by black lines and (ii) within sexes between proteins by blue and orange lines for men and women, respectively. Statistical significance was determined by 2-way ANOVA followed by multiple end-point correction. Significance levels are *$P \leq 0.05$; **$P \leq 0.005$; ***$P \leq 0.001$; ****$P \leq 0.0001$.

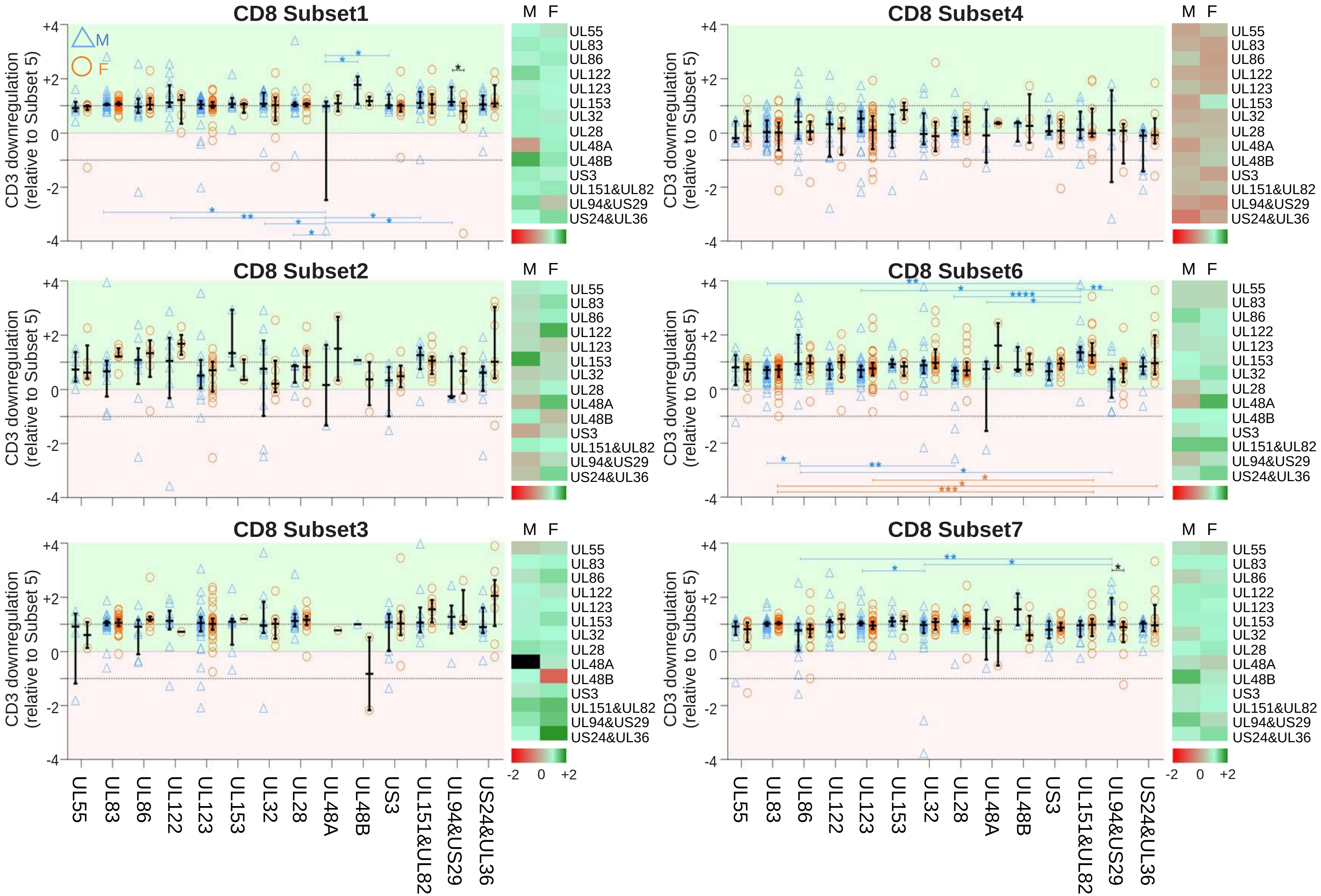

Figure S6. Analysis of CD3 downregulation in different functional CD8 T-cell subsets in women and men. PBMCs from men (n=47) and females (n=47) were stimulated with specific CMV-protein peptide mixes and processed for intracellular cytokine staining, excluding responses below the 1/10,000 threshold. Levels of CD3 downregulation in functional subsets (Sub1 through Sub7) were standardized relative to the downregulation in Sub5. Heatmaps show CD3 downmodulation in males (M) compared to females (F). Darker shades of red represent low and darker shades of green high CD3 downmodulation. Scatter plots show CD3 downregulation in males (blue triangles) and females (orange circles). Error bars show median and interquartile range. Statistically significant differences (i) between sexes are indicated by black lines and (ii) within sexes between proteins by blue and orange lines for men and women, respectively. Statistical significance was determined by 2-way ANOVA followed by multiple end-point correction. Significance levels are *$P \leq 0.05$; **$P \leq 0.005$; ***$P \leq 0.001$; ****$P \leq 0.0001$.

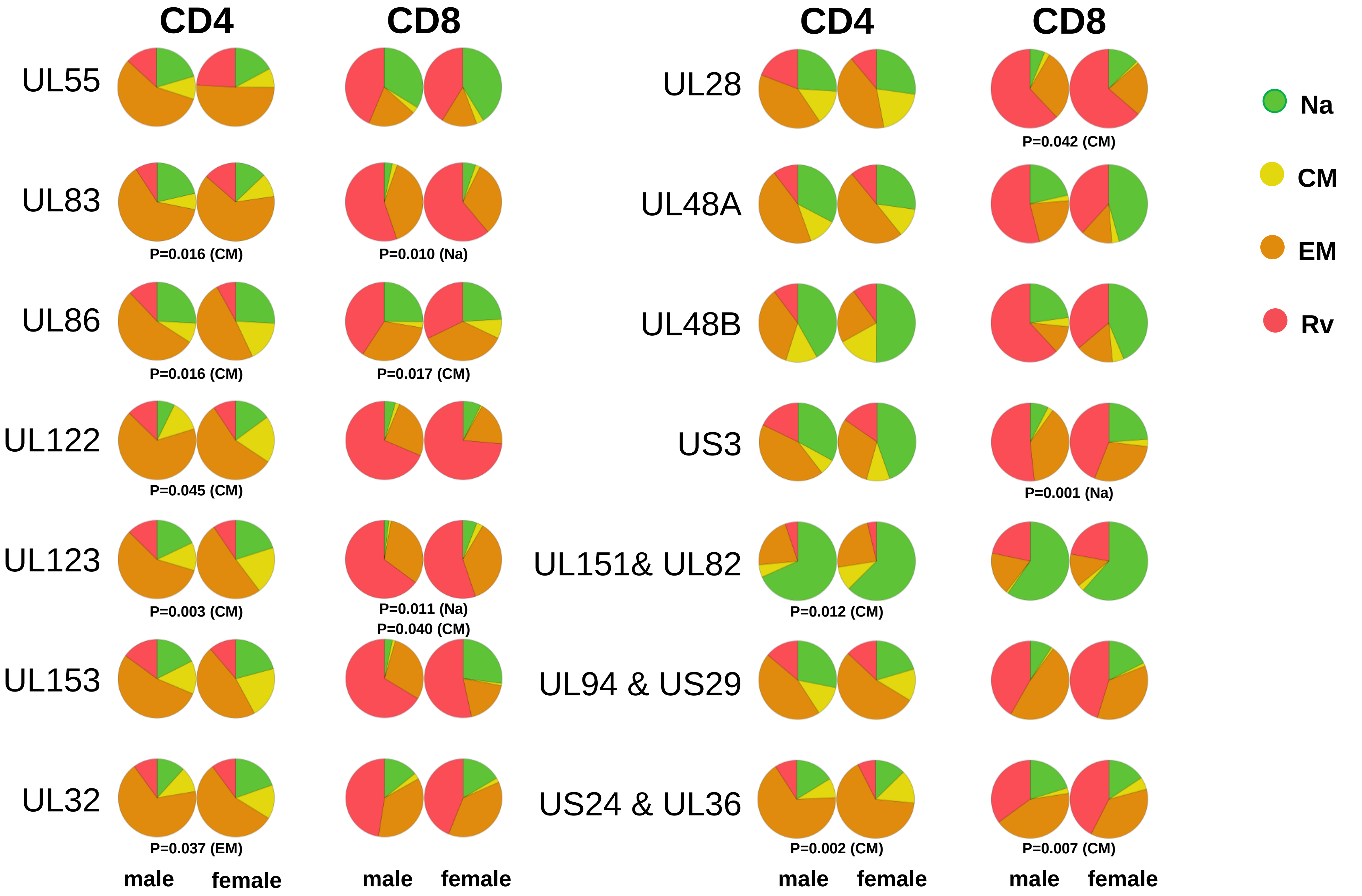

Figure S7. Distribution of CMV protein-specific T-cells across canonical memory T-cell compartments. Pie charts show the percentage of responding cells for each memory subset (pie slices) in CD4 and CD8 T-cells for males (left) and females (right). Each row corresponds to the response to one CMV peptide pool. *p*-values at the bottom of pie charts show significant ($p<0.05$) differences between males and females.

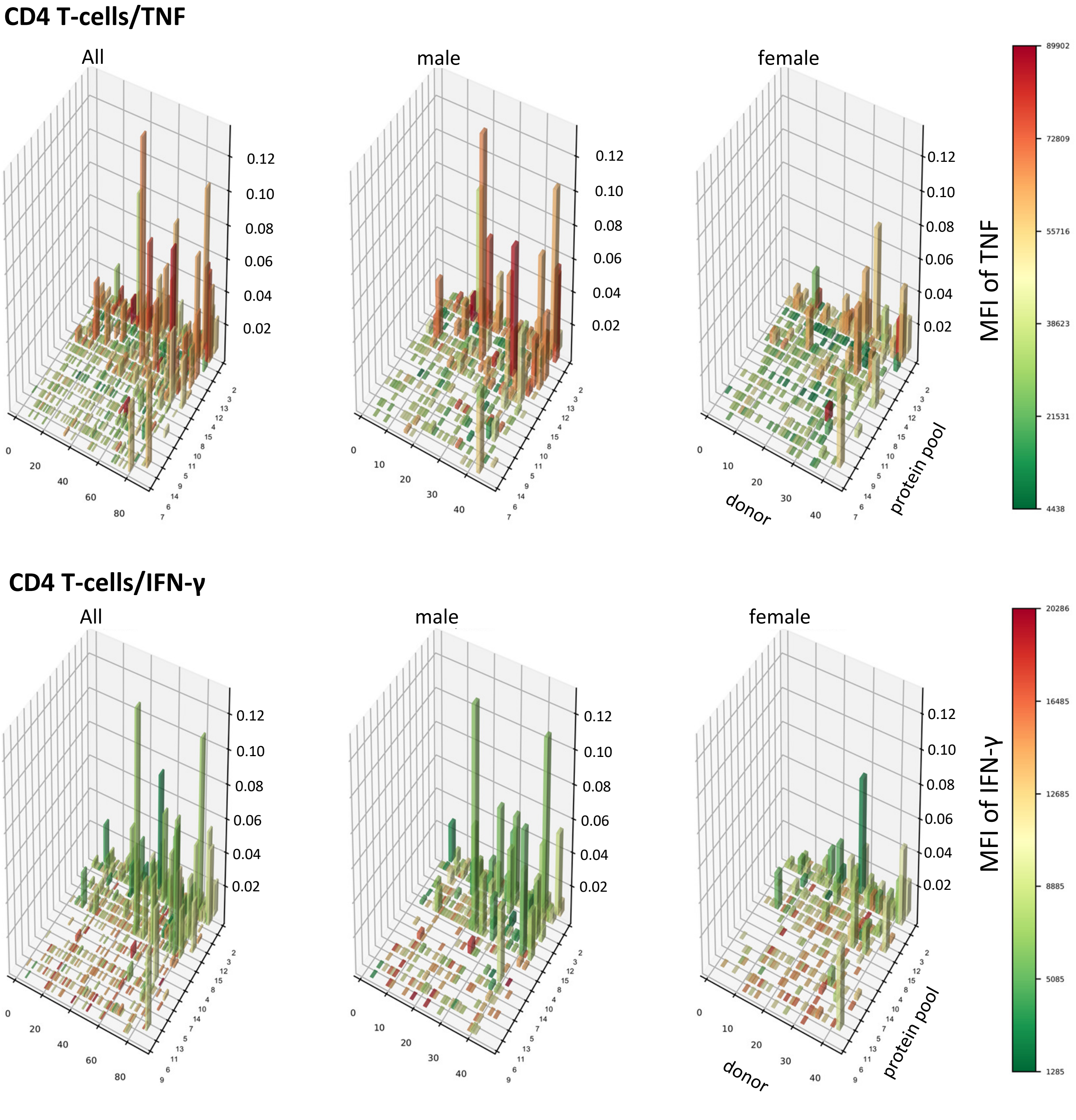

Figure S9. Large CD4 T-cell responses producing high levels of TNF are found preferentially in men. 3D bar charts show all individual CD4 T-cell responses to each CMV peptide pool in the entire population (left), men alone (middle) and women alone (right). Participants are ordered by increasing median T-cell response size from left to right with respect to the entire population. Peptide pools are ordered by increasing median response size induced from front to back, also with respect to the entire population. Bar height corresponds to the cell counts (cell/nL) of cytokine producing CD4 T-cells, i.e., response size. Bar colour corresponds to the MFI of the measured cytokine according to the displayed scale.

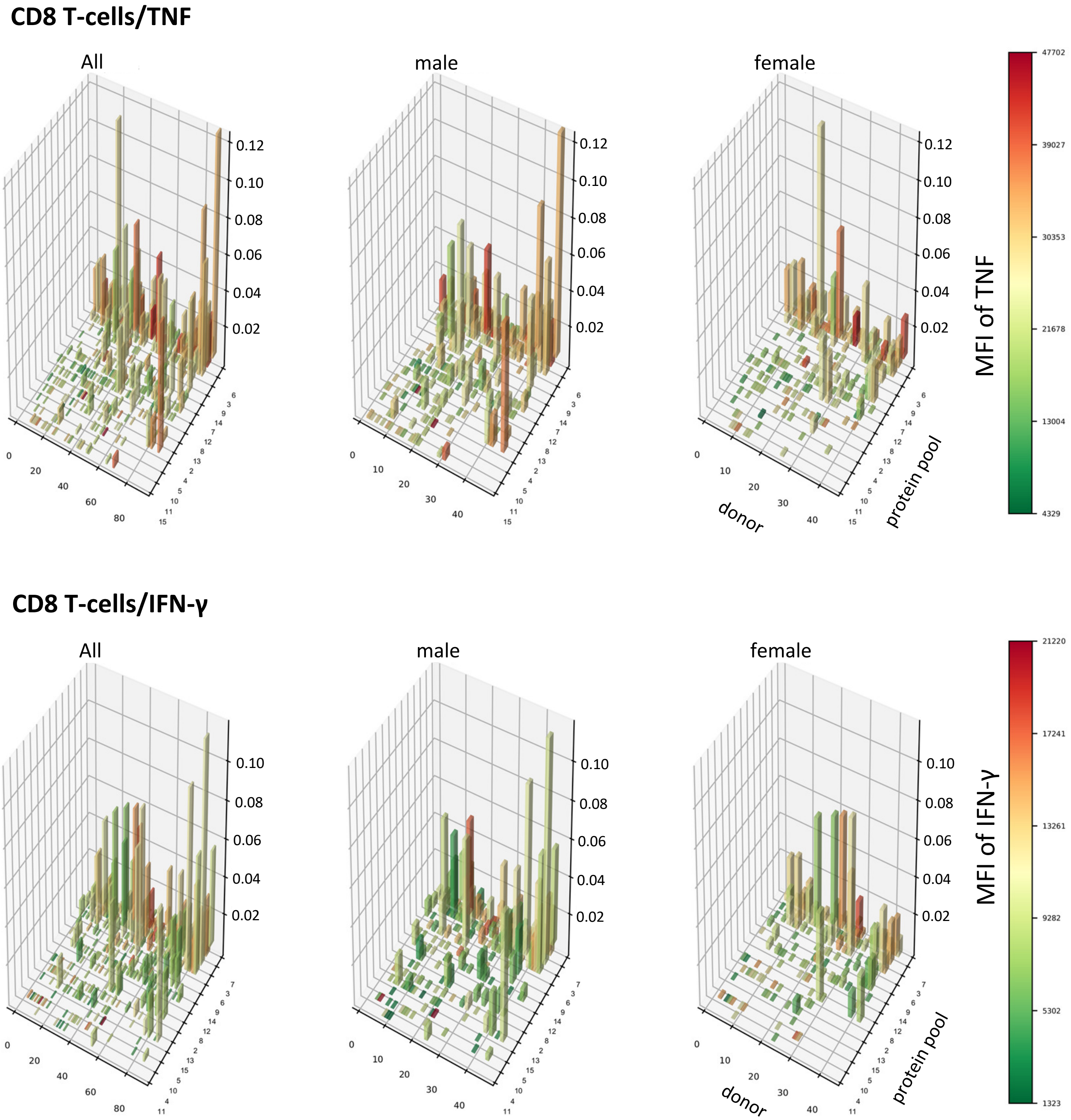

Figure S10. Large CD8 T-cell responses producing high levels of TNF are found preferentially in men. 3D bar charts show all individual CD8 T-cell responses to each CMV peptide pool in the entire population (left), men alone (middle) and women alone (right). Participants are ordered by increasing median T-cell response size from left to right with respect to the entire population. Peptide pools are ordered by increasing median response size induced from front to back, also with respect to the entire population. Bar height corresponds to the cell counts (cell/nL) of cytokine producing CD8 T-cells, i.e., response size. Bar colour corresponds to the MFI of the measured cytokine according to the displayed scale.

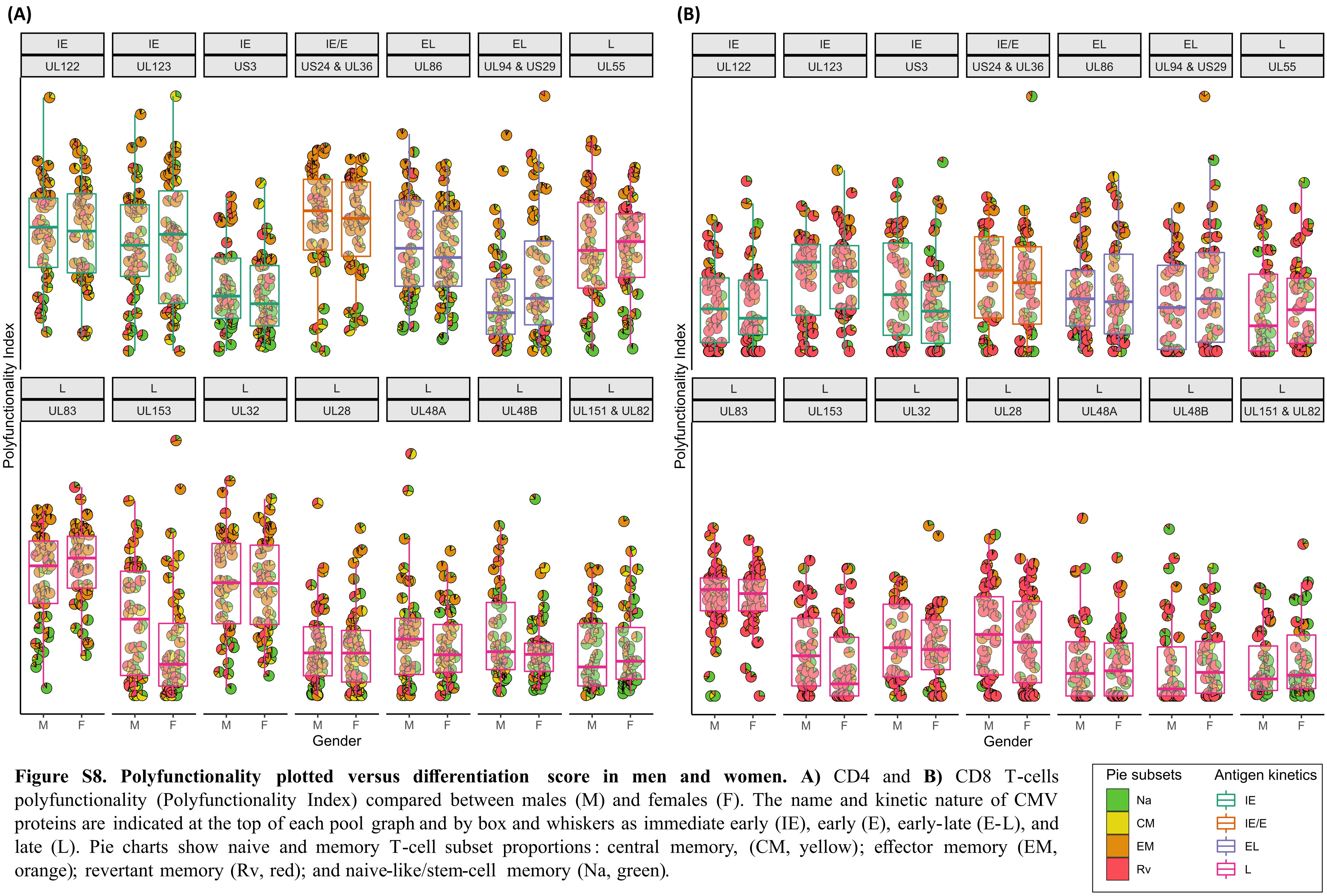

Figure S8. Polyfunctionality plotted versus differentiation score in men and women. A) CD4 and **B)** CD8 T-cells polyfunctionality (Polyfunctionality Index) compared between males (M) and females (F). The name and kinetic nature of CMV proteins are indicated at the top of each pool graph and by box and whiskers as immediate early (IE), early (E), early-late (E-L), and late (L). Pie charts show naive and memory T-cell subset proportions: central memory, (CM, yellow); effector memory (EM, orange); revertant memory (Rv, red); and naive-like/stem-cell memory (Na, green).

Table S1. Correlation between counts per volume of blood and percentages of CMV-specific T-cells

	CD4 T-cells			CD8 T-cells		
**Response size of reference T-cell population**	**IL-2 % vs cells/nL**	**TNF vs cells/nL**	**IFN-γ % vs cells/nL**	**IL-2 % vs cells/nL**	**TNF % vs cells/nL**	**IFN-γ % vs cells/nL**
**UL55**	0,954***	0,875***	0,877***	0,979***	0,978***	0,984***
**UL83 (‘pp65’)**	0,911***	0,918***	0,908***	0,940***	0,885***	0,882***
**UL86**	0,915***	0,952***	0,951***	0,999***	0,846***	0,747***
**UL122**	0,896***	0,847***	0,864***	0,993***	0,983***	0,980***
**UL123 (‘IE-1’)**	0,850***	0,983***	0,990***	0,869***	0,794***	0,747***
**UL153**	0,990***	1***	1***	0,915***	0,905***	0,900***
**UL32**	0,934***	0,945***	0,951***	0,535*	0,767***	0,773***
**UL28**	0,834***	0,929***	0,809***	0,748***	0,737***	0,823***
**UL48A**	0,933***	0,919***	0,923***	0,862*	0,913***	0,874**
**UL48B**	0,961***	0,988***	0,801***	0,992***	0,896***	0,996***
**US3**	0,921***	0,908***	0,911***	0,987***	0,964***	0,948***
**UL151& UL82**	0,817***	0,868***	0,974***	0,832***	0,936***	0,954***
**UL94 & US29**	0,881***	0,868***	0,981***	0,981***	0,991***	0,988***
**US24 & UL36**	0,907***	0,958***	0,964***	0,971***	0,975***	0,974***

Table S2. Protein recognition versus summated response size

**CD4 all CMV proteins R (N)**	**CD4 all CMV proteins**			
**CD8 all CMV proteins R (N)**	-0.003 (94)	**CD8 all CMV proteins**		
**CD4 no. of proteins recognized R (N)**	0.258 (94) (*p*=0.012)	-0.042 (94)	**CD4 no. of proteins recognized**	
**CD8 no. of proteins recognized R (N)**	0.081 (94)	0.115 (94)	0.571(97) (*p*=0.000)	**CD8 no. of proteins recognized**

Table S3. Statistical differences in response sizes. Impact of CMV protein and biological sex (2-way ANOVA test)

**CD4 T-cells**	**CMV protein pool (*p*)**	**Sex (*p*)**
IL-2	0.0003	0.0013
TNF	0.0001	0.0030
IFN-γ	≤0.0001	0.0097
**CD8 T-cells**		
IL-2	n.s.	n.s.
TNF	≤0.0001	0.0467
IFN-γ	≤0.0001	n.s.

Table S4. Functional subsets showing significant differences in CD3 downmodulation

	**CMV protein pool (*p*)**	**Sex (*p*)**
**Sub1**	0.0006	n.s.
**Sub3**	≤0.0001	0.0003
**Sub4**	0.001	0.0339
**Sub6**	≤0.0001	0.0137
**Sub7**	0.0001	0.0001
